# Enhancing tribo-mechanical, microstructural morphology, and corrosion performance of AZ91D-magnesium composites through the synergistic reinforcements of silicon nitride and waste glass powder

**DOI:** 10.1038/s41598-024-52804-y

**Published:** 2024-02-08

**Authors:** Shubham Sharma, Shashi Prakash Dwivedi, Abhinav Kumar, Fuad A. Awwad, M. Ijaz Khan, Emad A. A. Ismail

**Affiliations:** 1 Centre for Research Impact and Outcome, Chitkara University Institute of Engineering and Technology, Chitkara University, Rajpura, Punjab 140401 India; 2 School of Mechanical and Automotive Engineering, Qingdao University of Technology, Qingdao, 266520 China; 3 Department of Mechanical Engineering, Lebanese American University, Kraytem, Beirut, 1102-2801 Lebanon; 4 Faculty of Mechanical Engineering, Opole University of Technology, 45-758 Opole, Poland; 5 Department of Mechanical Engineering, Lloyd Institute of Engineering & Technology, Knowledge Park II, Greater Noida, Uttar Pradesh 201306 India; 6 Department of Nuclear and Renewable Energy, Ural Federal University Named After the First President of Russia, Boris Yeltsin, 19 Mira Street, Ekaterinburg, Russia 620002; 7 Department of Quantitative Analysis, College of Business Administration, King Saud University, P.O. Box 71115, 11587 Riyadh, Saudi Arabia; 8 Department of Mechanics and Engineering Science, Peking University, Beijing, 100871 China

**Keywords:** Engineering, Materials science

## Abstract

The present investigation has employed recycled waste glass powder (WGP) and silicon nitride (Si_3_N_4_) as reinforcing-agents within AZ91D-matrix composites. The composites were fabricated by employing the vacuum stir casting technique to mitigate the effects of oxidation and to ensure homogeneity, uniformity, and superior wettability among the AZ91D-matrix and reinforcements. A microscopic study provided confirmation of a uniform dispersion of WGP and Si_3_N_4_ particles throughout the AZ91D-matrix. The tensile strength of the AZ91D/WGP/Si_3_N_4_ composites rise with the inclusion of WGP particulates by up to 1.5 percent in AZ91D/7.5% Si_3_N_4_. However, the tensile strength of the AZ91D/9%Si_3_N_4_ composite have showed maximum value as compared to other chosen formulations/combinations in the current investigation. The tensile strength of AZ91D/1.5% WGP/7.5% Si_3_N_4_ composites has strengthened up to 12.13 percent with the comparison of base alloy AZ91D-matrix. In A1 formulated composite, the amount of WGP particulate has enhanced the hardness of the AZ91D-alloy by up to 1.5 percent. Findings, nevertheless has exhibited that the A6 formulated composite had superior outcomes in terms of hardness. The incorporation of “reinforcing-constituent particulates” with 1.5%WGP + 7.5%Si_3_N_4_ combination within the AZ91D-matrix, has further increased fatigue-strength by around 57.84 percent. A weight-loss of 0.312 mg was being unveiled for the A1 formulated fabricated composite. The weight-loss for the A6 formulated fabricated composite, however, was reported to be 0.294 mg. At 5 N loads, 2 m/s sliding speed, and 1000 m of sliding distance, the developed 1.5%WGP/7.5%Si_3_N_4_/AZ91D composites was reported to have a rate of wear, and frictional coefficient of 0.0025 mm^3^/m and 0.315, respectively. The investigation employing scanning electron microscopy (SEM) identified the presence of corrosion pits on the surfaces that had undergone corrosion. These pits were found to be a result of localised surface assaults occurring in corrosive environments. Additionally, SEM pictures of the worn surfaces indicated the emergence of microcracks, which may be associated to the conditions of cyclic loading. Moreover, the tensile-fractography examination for the developed 1.5%WGP/7.5%Si_3_N_4_/AZ91D composites has exhibited the brittle fracture failure, including cracks and debonding phenomena. In addition, the EDS spectra-analysis have revealed an apparent existence of the observed Mg-peak, Si-peak, Al-peak, Ca-peak, and O-peak for the 1.5%WGP/7.5%Si_3_N_4_/AZ91D composites. Furthermore, the utilisation of X-ray diffraction analysis effectively determined the existence of hard phases inside the AZ91D-matrix, which significantly contributed to the reported enhancement in wear resistance. The development of harder-phases has included, α-Mg, Al_12_Mg_17,_ SiO_2_, Si_3_N_4_, MgO, and CaO phases within the composite has been accountable for the enhancement of the tribomechanical, and wear-resistance characteristics of the AZ91D/WGP/Si_3_N_4_ composites. The Si_3_N_4_ has been discovered to have a substantial impact on enhancing mechanical performance and raising the resistance to wear.

## Introduction

The wetting behavior between ceramic particles and magnesium alloy is an important property to consider during casting processes in order to control the flow and distribution of the particles within the magnesium matrix. Ceramic particles, such as SiC, Si_3_N_4,_ TiB_2_ etc. are commonly added to magnesium alloys to improve their mechanical characteristics, including hardness, wear resistance, and thermal stability. However, the effective reinforcement of the ceramic particles within the magnesium matrix depends greatly on their wetting behavior. Wettability between ceramic particles and the magnesium alloy can be affected by several key factors, such as surface energy, interfacial chemistry, and surface roughness. The surface energy of the ceramic particles is generally higher than that of the magnesium alloy, resulting in poor wetting behavior^1^. This can be improved by modifying the surface chemistry of the ceramic particles through chemical treatments or by using surfactants to reduce the surface tension between the ceramic particles and the magnesium alloy. Furthermore, the surface roughness of the ceramic particles and magnesium alloy can also influence their wettability. The presence of rough or uneven surfaces can lead to poor wetting behavior due to the development of a barrier coating between the particles and the alloy. This can be addressed by pre-treatment of the surfaces or by adjusting the casting parameters to ensure a more favorable contact angle. Effective dispersion of ceramic particles within the magnesium alloy is critical for achieving the desired mechanical properties. Therefore, techniques such as ultrasound assisted dispersion, stir casting, and rheo-casting have been developed to improve the wettability of the ceramic particles within the magnesium alloy. Additionally, the use of coupling agents and adhesion promoters can further enhance the interfacial adhesion between ceramic particles and magnesium alloy^2–4^.

The investigator must implement his concept in order to use engineering materials. To meet a variety of performance needs, novel materials like composites are currently being developed. The primary driver of the rise of magnesium-based composite materials is the requirement for certain applications for materials with higher elastic modulus, impact endurance, tension strength, and hardness than typical magnesium alloys. The resistance to wear for traditional magnesium alloys is likewise relatively low. Composite construction materials have succeeded in replacing conventional engineering materials^3^. Due to their superior mechanical characteristics, outstanding durability against wear, lightweight, and ability to withstand corrosion at high raised temperatures, composite substances are particularly favoured. A composite material is made by mixing two or more various substances, each one of which has distinct properties, to produce an entirely novel material with properties that the individual components are unable to achieve. Two or more different materials, however, retain their unique characteristics while also passing on their attributes to new materials. In order to improve the overall characteristics of the matrix, reinforcement of some form is typically integrated with the continuous big phase (matrix) in tiny quantities^4^.

The kind, quantity, and size of reinforcing particles have a significant impact on the structural integrity of composite materials. The strength of the composite increases with the degree of dispersed phases. Contrasting to reinforcement, which is typically a hard and strong material, matrix is typically a flexible and tough structure^5^. The qualities of both the matrix and the reinforcement are combined in the composite to form a material that has attributes that are superior than both the matrix and the reinforcement^6^ if the composite is properly designed, customized, and built using the right manufacturing procedure.

The structural metal magnesium, which has a less dense structure, is combined with other metals like Zr, Cu, Si, Mn, Zn, Al, and other rare-earth metals to form Mg alloys. Magnesium alloys have a hexagonal lattice pattern and affect the fundamental characteristics. Compared to cubic latticed materials like steel, Cu, and Al, hexagonal lattice exhibiting plastic distortion is significantly more complex. As a result, cast alloys made of Mg are common. Cast magnesium alloys are used in the manufacture of numerous components, including those used in lenses, camera bodies, high-performance car parts, and parts for contemporary automobiles. Nearly majority of the industrial magnesium alloys developed in the United States (US) comprise aluminium (3–13%), manganese (0.1–0.4%), and zinc (0.5–3%), respectively. Sand castings are done with AZ92 and AZ63, while permanent mould castings are done using AZ92 most of the time (although A10 and AZ63 can be utilised as well in the latter equipment). For applicability in “die castings” is AZ91 alloy. The majority of the instances, alloy-AZ61 is employed for forgings, where AZ80-alloy is utilised for the “highest-strength”, and “M1 for lower-strength”^8–10^.

By using electromagnetic stirring, Zhang Xiao-li et al.^7^ fabricated the magnesium AZ91. A distinctive microstructure was observed. The rupturing surface morphology of tensile specimens is dependent on the solid percentage of the original particles, as demonstrated by the fractographic microstructures. During the extrusion procedure of ZK60 alloys, finer as well as more homogeneous microstructure and enhanced mechanical characteristics were demonstrated by Yu et al.^8^. Employing electromagnetic stir casting, T. Y. Kwak et al.^9^ developed an “AZ80 alloy-based composites” reinforced with “1 wt% Ca (1 wt% Ca-AZ80 alloy)”. To investigate the “hot-deformation behaviour” of the casted, “1 wt% Ca-AZ80 alloy”, compression experiments were performed. The “temperature”, and “strain-rate” ranges for the compression test were 523–693 K and 10^−3^ to 10^1^ s^−1^, respectively. At strains of 0.2–0.5 s^−1^, the “processing-maps” of the casted, “1 wt% Ca-AZ80 alloy” were compared to those of the “casted AZ80-alloy” that had been processed conventionally. When the electromagnetic field’s frequency is kept low during low-frequency electromagnetic casting, forced convection happens in the melt. The induced convection resulted in a uniform temperature field and solidification velocity. The examination of the results led to the conclusion that the heat transfer behaviour is governed by electromagnetic frequency, casting temperature, and stirring current^10,11^. Moreover, the reinforcing-additives have contributed a crucial role behind raising the characteristics of composites.

Silicon nitride (Si_3_N_4_) as particulates is a class of ceramics which seek widespread applicability in multiple-sectors. The superior thermomechanical, and chemical characteristics of Si_3_N_4_ particulates render them viable for application across numerous-sectors. Their remarkable resistance to stress caused by heat, thermal-shock, thermal-damage, thermal-stresses caused by elevated temp., their higher strength, and hardness has provided them with the ability to withstand severe harsh environmental-conditions. In order to further enhance the mechanical characteristics of composites, Si_3_N_4_ particulates have been utilised as reinforcing-additives. In the automobile, aviation, and cutting-tool or machine-tool sectors, among others, Si_3_N_4_ particulates have been employed as a result of their insulating characteristics, thermal-barrier and resistance to wear. Furthermore, their electrically insulation characteristics rendering Si_3_N_4_ particulates valuable in electronically as well as semiconductor-like applications.

As the glass-cullet, or WGP, is a by-product of glass-manufacturing. It is produced through the pulverisation of broken waste-glass into granules or finely-powdered form, which can be utilised as a raw-material in the manufacturing of newly developed glass-products. In addition, WGP has been employed within the construction industry as an alternative substitute for materials including cement, sand, and other construction-applications as aggregates. WGP has served as a sustainable, environmentally-friendly replacement for conventional materials owing to its potential to minimise waste generated by landfills and eliminate the necessity for the new raw materials. Additionally, it exhibits the ability to mitigate carbon dioxide emissions, greenhouse-gases, carbon-footprints, and airborne-pollutants by virtue of its energy-effective production techniques, which demand lesser energy to produce WGP than those that generate virgin materials. WGP has offered an extensive array of technological upsides, in addition to its environmental excellence. Chemically-stable, along with possessing a lower coefficient of thermal expansion, it is suitable for usage in the production of ceramics made from glass. Additionally, owing to its nonreactivity, WGP can be employed as a filling additive in adhesives, protective coatings, and composites.

In context with the problem formulation, the focus of this investigation is to strengthen the tribomechanical characteristics of AZ91D magnesium-based composites. The primary underlying intention is the utilisation of WGP and silicon nitride (Si_3_N_4_) as reinforcing agents in order to enhance the corrosion resistance, resistance to abrasion, and mechanical characteristics of the composites. The focus for this research is to analyse the implications of varying weight percentages for WGP and Si_3_N_4_ on the composites’ microstructure morphology, mechanical characteristics, corrosion resistance, and tribological performance. Although, by achieving a consistent even dispersion and uniformly homogenous-distribution of such reinforcing-constituent agents within the AZ91D-matrix offers a significant challenge, provided the concerns encircling oxidation that occur throughout the manufacturing method. All in all, by employing vacuum stir casting for developing composites with enhanced characteristics while minimising the implications concerning oxidation was the intent of this research.

Moreover, the primary focus that is being investigated pertains to the necessity for strengthening the mechanical as well as wear-resistant characteristics of AZ91D magnesium alloy, particularly under circumstances involving cyclic-loading as well as corrosive-surroundings.

As far as research gaps are concerned, the comprehensive analysis on the synergistic influences of WGP and Si_3_N_4_ in AZ91D composites is limited in the existing literature. The currently existing knowledge voids pertaining to the inadequate research into microstructural characteristics, the implications of various reinforcing proportions, as well as an in-depth investigation of the mechanisms underlying wear and corrosion resistance for these developed composite materials.

Although the study examines the incorporation of WGP and Si_3_N_4_, there are certain aspects of the existing scholarly scientific literature that have not been thoroughly researched. The reinforcing particulate of magnesium-based composites have been the focus of prior studies; however, the synergistic impacts of WGP and Si_3_N_4_ on AZ91D alloy remained unexplored. Furthermore, there is an absence of studies addressing the impact of these reinforcements on corrosion resistance as well as a thorough investigation of microstructural morphology variations caused by varying compositions. Moreover, the intent for this research being conducted is to address this gap by means of an examination regarding the synergistic combined impacts of all these reinforcement materials on the composites’ microstructure morphology, mechanical strength, resistance to wear, and corrosion behaviour. For instance, the interaction-relationship, bonding-strength, wettability, and adhesion-strength of such reinforcing agents with the AZ91D-matrix, the influence of fabrication variables on the physicomechanical characteristics of the composite, and an in-depth comprehension of the tribo-corrosion mechanisms in magnesium-based composites have not been thoroughly explored.

Although prior study has focused on magnesium-based composites, the synergistic impact for recycled WGP as well as silicon nitride on the microstructure morphology, mechanical characteristics, wear resistance, and corrosion behaviour of AZ91D magnesium-based composites remains unresolved. Additional research is required to ascertain the precise contributions of WGP and Si_3_N_4_, as well as how they are distributed as well as the subsequent impacts on numerous characteristics. Prior investigations may have disregarded the necessity of performing a thorough examination of the XRD characteristics of the developed composites as well as the underlying processes that are responsible for the observable enhancements.

In accordance with research goals are concerned, following are the intended objectives, firstly, the intent is to optimise the proportion of weight of WGP and Si_3_N_4_ in order to attain enhanced mechanical and wear characteristics of the composite. Secondly, by utilising SEM analysis as well as optical microscopy for examining the distribution of WGP and Si_3_N_4_ reinforcing-particulates within the AZ91D-matrix for the intent of microstructural analysis. Comprehend the parameters that are influencing the dispersion. Thirdly, to conduct an assessment concerning the fatigue strength, modulus, ductility, tensile strength, as well as hardness of the composites that have been developed. Determine the optimal WGP as well as Si_3_N_4_ blend combination as reinforcing-particulates for strengthening the mechanical characteristics. Then to evaluate the composites’ resistance to corrosion in a corrosive environment comprising 3.5% NaCl. Analyse the interaction-correlation among the formation of corrosion-pits, as well as the existence of WGP and Si_3_N_4_ reinforcing-particulates. Afterwards, to employ Pin-on-Disc testing for evaluating the wear-rates, and coefficient of friction characteristics of the distinct composite formulations to determine the optimal blend combination of reinforcements. Analyse the implications of WGP and Si_3_N_4_ reinforcing-particulates on the enhancement of resistance to wear. Finally, to determine the phases contained (α-Mg, Al_12_Mg_17_, SiO_2_, Si_3_N_4_, MgO, CaO) and ascertain how they contributed to the enhanced characteristics through analysing the XRD structure patterns of the composites.

All in all, the investigation seeks to examine the implications of WGP and Si_3_N_4_ on AZ91D magnesium-based composites pertaining to their microstructure, physicomechanical characteristics, wear resistance, and corrosion behaviour. The specific aims comprise the following aspects: accomplishing or attaining an even homogenous distribution of reinforcing-agents, determining the mechanical characteristics, exploring the resistance of the developed composites against corrosion, and comprehending their worn behaviour characteristics.

Concerning the scope and significance of the present study, the scope of this research has entailed the development for composites comprising various proportions of WGP and Si3N4, as well as an in-depth analysis of their microstructure morphology, and characteristics. The prospective enhancement for the performance functioning, effectiveness, or efficiency for AZ91D magnesium alloys is of profound paramount significance as it allows or facilitates the possible expansion of their potential applications in industries requiring superior mechanical and corrosion characteristics. The composites’ prospective applications in sectors including aviation along with automobiles, that require lightweight materials with remarkable mechanical as well as wear characteristics, are of considerable importance.

Additionally, the aim of the research comprises the manufacturing of advanced AZ91D composites that possess customised characteristics, with the intention of employing them for sectors requiring composites with superior high strength performance.

Moreover, in terms of scientific originality, this research’s novelty is derived from its investigation into the implications of combining WGP and Si_3_N_4_ as reinforcing constituent particulates on the microstructural morphology, and characteristics of AZ91D composites. A novel aspect has been incorporated to the current scientific literature on magnesium composites resulting from the synergistic blend combinated effect of these materials, their evenly homogenous arranged, and uniformity of distribution within the AZ91D-matrix, along with assessing their implications on mechanical and wear characteristics. Furthermore, the research offers a substantial valuable contribution to the scholarly literature pertaining to corrosion as well as wear processes in magnesium-based composites.

In reference to the scientific uniqueness, the unique distinctive characteristic lays in the blend combination of Si_3_N_4_, noted due to its thermodynamic stability, and WGP, a recycled material. The novel employment of these materials simultaneously in magnesium composites enhances insight pertaining their combined synergistic effects on the characteristics of the composite. The comprehensive examination regarding the manufacturing technique, microstructural morphological analysis, and the utilisation of Si3N4 as a reinforcing agent comprise the novel aspects. Additionally, the distinctive uniqueness of this research is the utilisation of SEM analysis to explore wear behaviour as well as evaluate the interfacial adhesion, and bonding-strength of reinforcing particulates. Moreover, to validate the reliability and precision of the findings, this study employed rigorous scientific techniques, including XRD analysis, mechanical testing, microscopic examination, and tribo-corrosion evaluation. Aiding to the rigorousness of this research, Optical Microscopy, SEM, and XRD analysis have been employed to conduct an in-depth assessment of the microstructure morphology, as well as phases that have been formed.

Finally, as far as the practical implications are concerned, the study’s findings have substantial implications in practice for sectors that rely on lightweight materials that exhibit enhanced mechanical and wear characteristics. The enhanced tensile strength, hardness, and abrasion resistance of the developed composites could potentially make them valuable for the manufacture of automobile as well as aircraft components, as well as additional structural components where these characteristics are critical. Furthermore, having insight into the material’s selection procedure for environments that are corrosive is a real world application-oriented advantageous effects of understanding of corrosion behaviour. The outcomes outline a scientific foundation for the prospective utilisation of Si_3_N_4_ as well as recycled WGP throughout the fabrication of advanced magnesium composites that have practical significance across numerous sectors. The practical implications for the developed composites entail the potential of employing them in real-world circumstances that require enhanced resistance to corrosion, wear, as well as mechanical strength. The findings from this investigation might yield valuable insights for industries that make use of magnesium-based alloys, whereas additionally offering direction for future research and development in the field of composite materials.

Furthermore, the incorporation of recycled WGP into the manufacturing of materials conforms with sustainable principles, environmentally friendly practices/standards, and makes a contribution to broader global environmental concern.

According to a thorough assessment of the literature, AZ91D has been used extensively in the development of MMCs and hybrid composites with typical ceramic reinforcing particles. However, only a small amount of research has been done employing “silicon nitride”, and “waste glass powder” (WGP) as reinforcement in the AZ91D-matrix. This feature is taken into consideration in the current research, which aims to build an AZ91D-based composite using silicon nitride as reinforcement. The inclusion of WGP and silicon nitride as “reinforcing particulate constituents” in the AZ91D-matrix material was identified by employing microstructural morphology, wettability, mechanical characteristics, corrosion, tribological, wettability, and XRD investigations.

## Experiments: materials and techniques

### Matrix material

**Figure 1:**
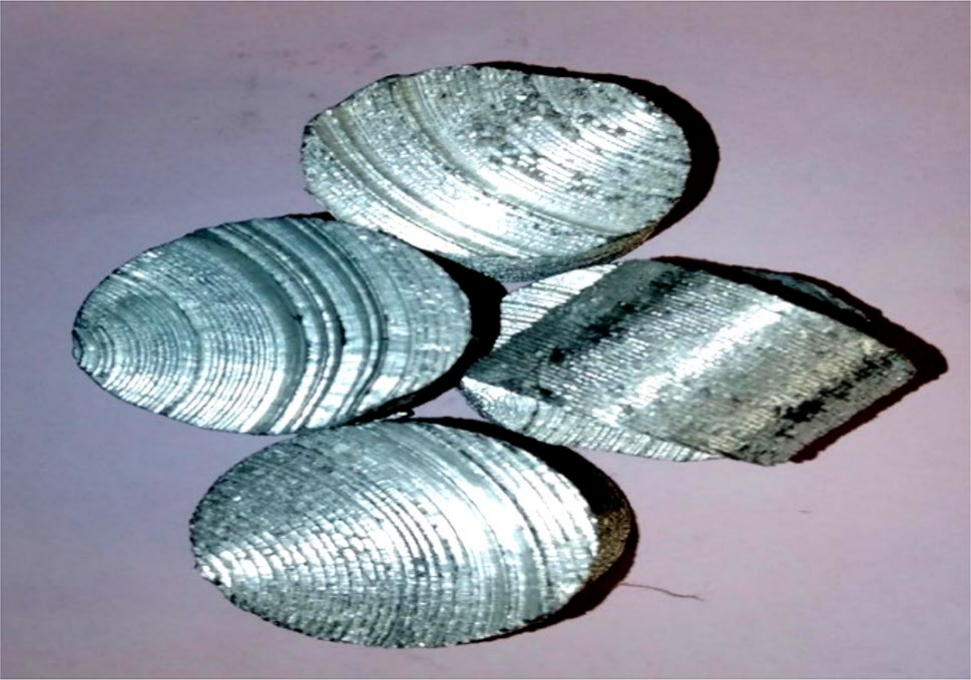
AZ91D Magnesium cast alloy.

**Figure 2:**
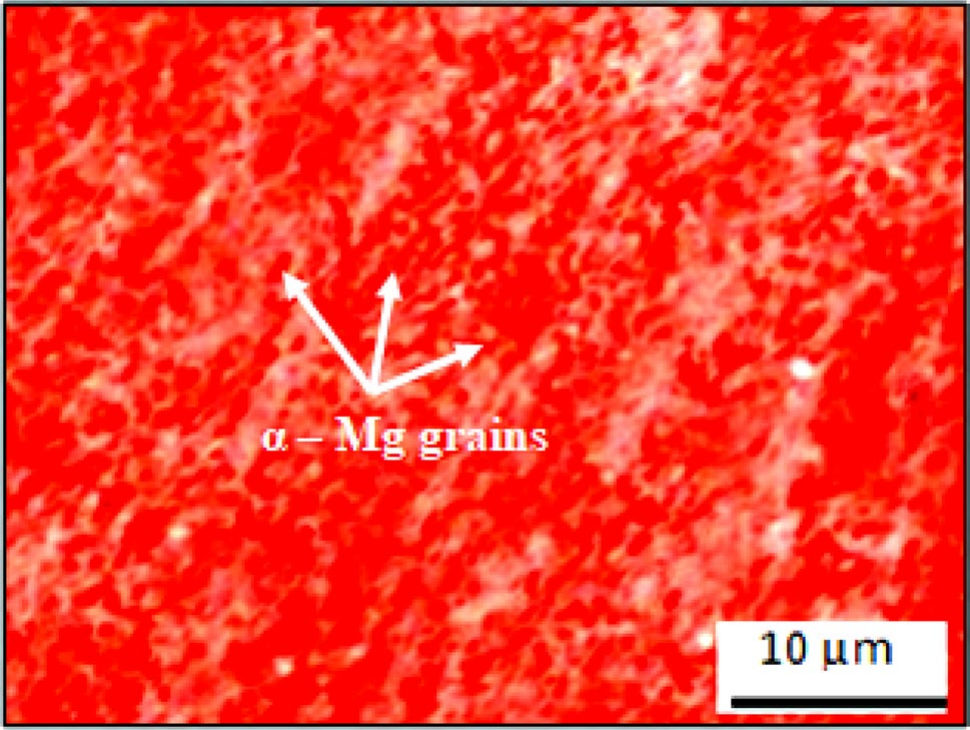
Optical microscopic images of “AZ91D Magnesium cast-alloy” without blending of reinforcing-particulates exhibiting α-Mg grains.

The matrix-material employed in this work is AZ91D-alloy (Fig. 1) with percent-elemental constituents as exhibited in Table 1. A high-purity-alloy with superior strength, great castability, and exceptional resistant to corrosion is AZ91D-Mg casted-alloy. It is mainly utilised in die-casting alloy. In order to strengthen the physicomechanical as well as wear characteristics for the composites, the reinforcement particulates have been incorporated to the matrix material, which functions as the base-material. AZ91D Mg-matrix is well-known for its superior resistance to corrosion, strength, durability, resilience, stability, toughness, and castability. Automobile components, aviation structures, and light-weight applications where the blend combination of minimal density as well as considerable excellent strength is crucial render significant widespread utilisation of AZ91D Mg-cast alloy. The grain-structure of α-Mg is discernible through microscopic examination, and variations in granular-size and shape could potentially reveal distinctive solidification circumstances or subsequent post-processing techniques. In addition, numerous items, including those for automobiles, household appliances, mobile phones, computers, sports equipment, handheld instruments, housings, and coverings, employ the AZ91D magnesium cast alloy based on the myriads of characteristics as illustrated in Table 2. The optical microscopic pictures of the “AZ91D-Mg casted-alloy” despite the incorporation of reinforcing particulates has been exhibited in Fig. 2. AZ91D magnesium cast alloy optical microscopic images allow one to see the α-Mg grain structure. Depending on how the grains are oriented in relation to the incident light, the boundaries among individual α-Mg grains can be bright or dark. These boundaries serve as a virtuous indication of the α-Mg grain structure. The separation among grain boundaries in the optical images can be employed to compute the size of the α-Mg grains. There will be lesser grain boundaries around regions with larger grains. Variations in grain size and shape may signify to various solidification circumstances or post-processing measures.

**Table 1:** Chemical constituents of the cast alloy of magnesium AZ91D.

Element	Ni	Fe	Cu	Si	Zn	Mn	Al	Mg	Others
Content (wt. %)	0.002	0.005	0.03	0.1	0.35–1	0.15–0.50	8.3–9.7	Remainder	0.02

**Table 2:** Mechanical characteristics of AZ91D magnesium cast alloy.

Properties	Hardness	Elongation/Ductility	Fatigue strength (10^8^ cycles)	Yield strength	Tensile strength	Density
Value	60 HV	7%	49.7 MPa	159 MPa	240 MPa	1.81 g/cm^3^

**Figure 3:**
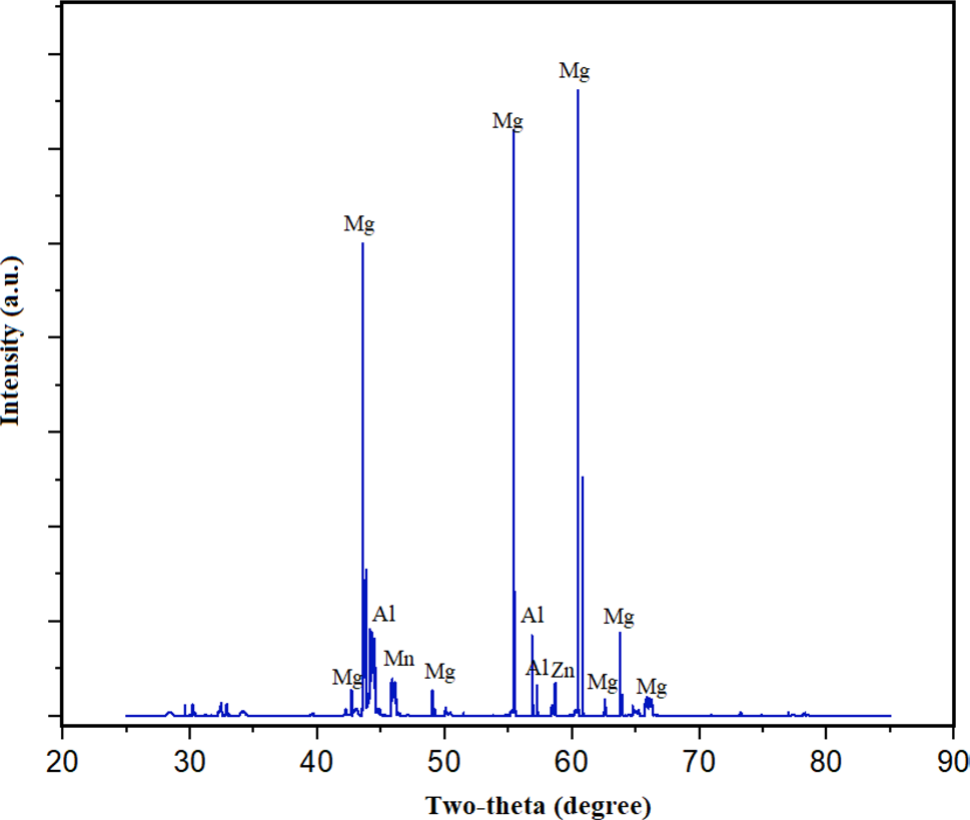
XRD of AZ91D Magnesium cast-alloy.

From the Fig. 3, the XRD pattern for AZ91D Magnesium cast-alloy has comprised of Mg, Al, and Zn, phases. The AZ91D-alloy has primarily comprised of alpha Mg-phase. Alpha Mg-phase is possessing the hexagonally close-packed crystal-structure. This phase has served as a pivotal role in escalating the alloy’s formability as well as ductility characteristics. The second phase is, Beta (β) Phase (Mg_17_Al_12_), as within the alloy, there is an aluminum-rich phase. This phase is having an orthorhombic crystal-structure. This phase has strengthened the alloy’s hardness, strength, and furthermore enhances its durability. The third detected phase is, Gamma (γ) Phase (MgZn_2_) as this phase has comprised of a significant amount of zinc. Here, the crystal-structure is cubic. This phase has contributed to the enhancement of the creep-resistance of the alloy or alloy’s resistant to creep-deformation. The degree to which the structure of a material is orderly-structured in a repeating three-dimensional pattern is referred to as its crystallinity. XRD has been employed for AZ91D-alloy to determine the specific crystal-arrangements, structures of crystals, and crystalline-patterns which are related to every phase. The degree of crystallinity serves as an indicator of the material’s degree of crystal structure^12,13^. Higher percentages of crystallinity reveal an additional orderly arranged atomic structure^14^. An XRD analysis of AZ91D-alloy can reveal how crystalline it is. This enhanced absorbing-intensity at a specific two-theta angle has indicated a higher-percentage of the corresponding planes of crystallography. Crystallinity variances in AZ91D-alloy may be caused by variables including casting-circumstances and thermal-processing. The measurement for crystallinity might be supported by XRD analysis of the intensity of diffraction patterns peaks. By identifying particular phases and analysing their corresponding concentrations that require absorption intensity at two theta angles. By employing the characteristic locations, orientations, and intensities of the diffraction peak patterns for every phase, it is possible to ascertain the phases that have been identified in the alloy as well as their corresponding proportions.

### Reinforcements: primary reinforcement material

**Figure 4:**
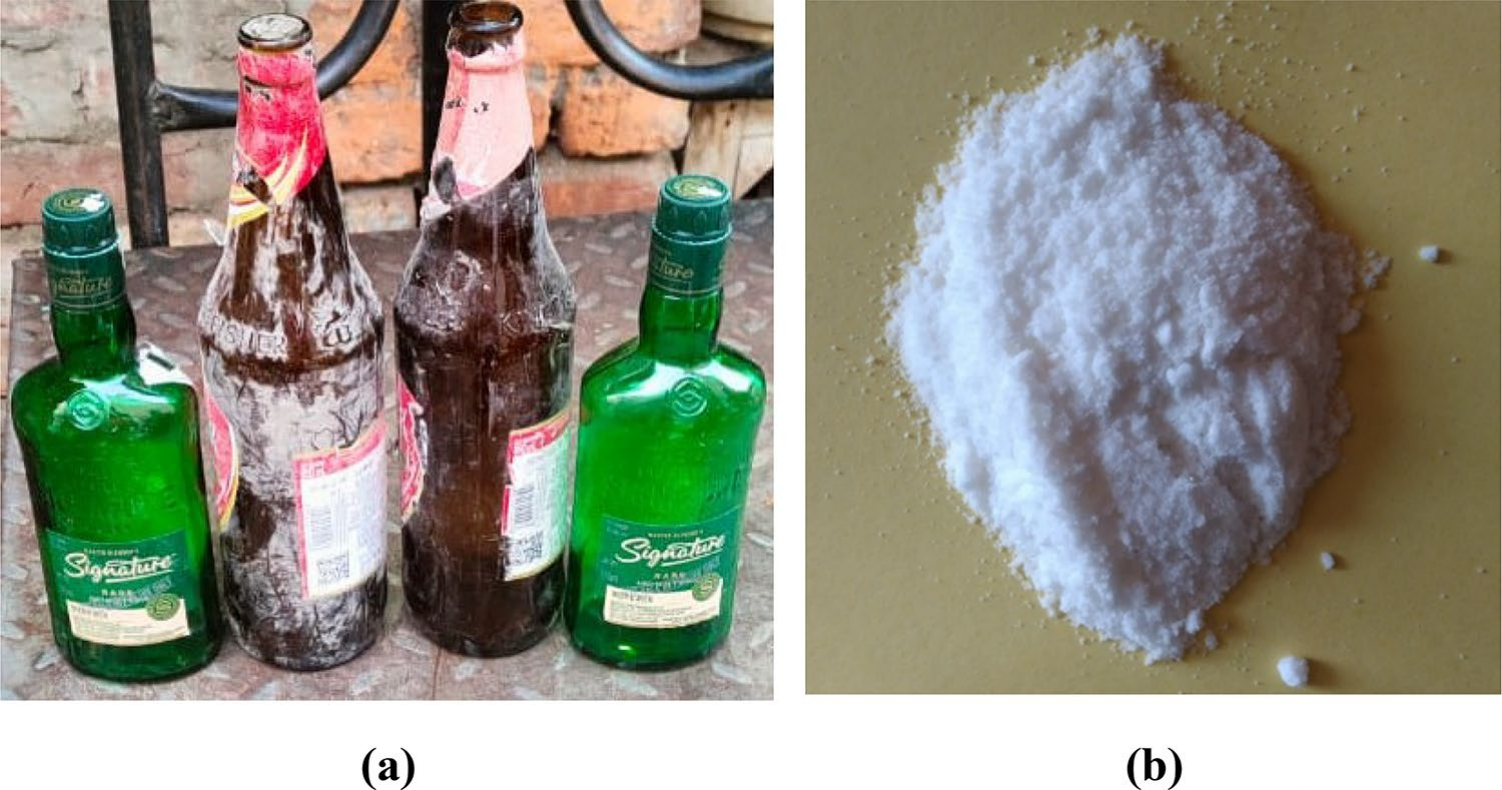
Illustration of (**a**) Waste-glass, (**b**) WGP particulates.

**Figure 5:**
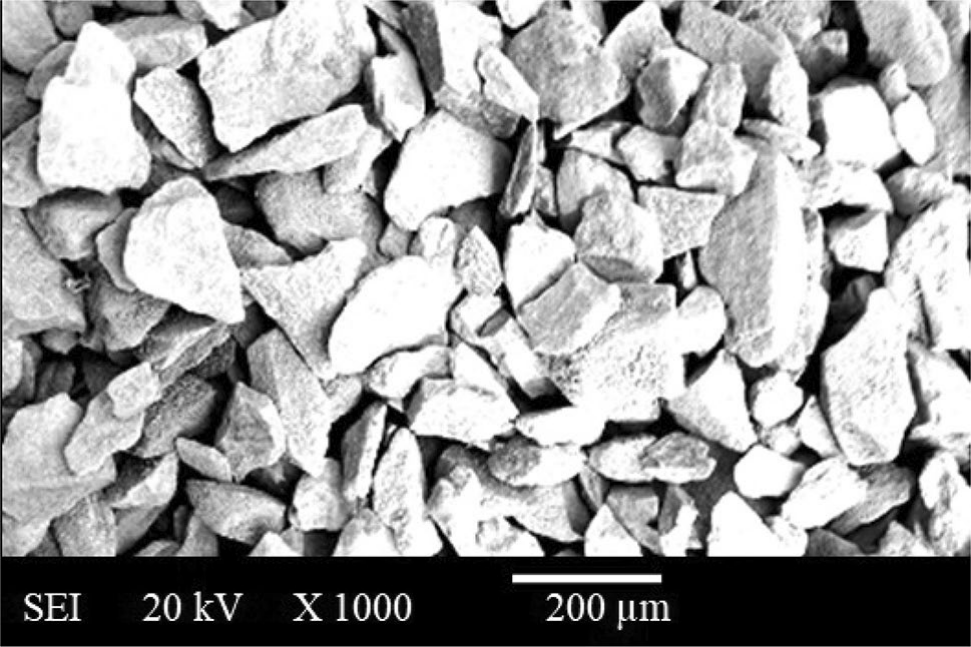
SEM image of WGP reinforcing-particulates.

**Figure 6:**
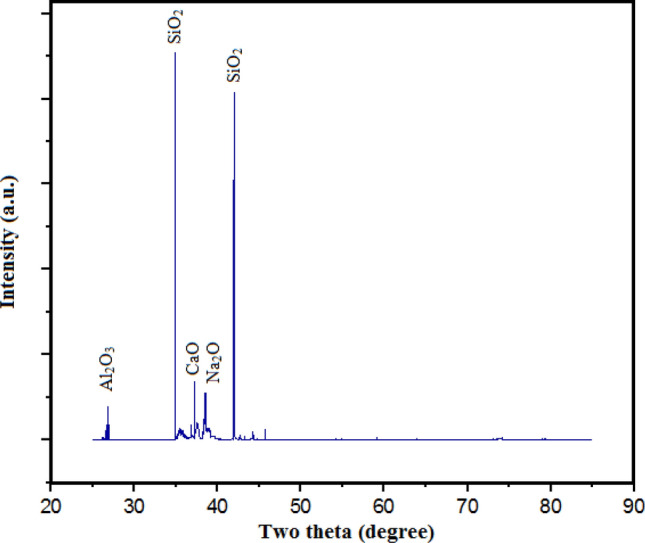
Powder-XRD of WGP particulate.

Locally available WGP as portrayed in Fig. 4a,b was collected or extracted, rinsed, crushed or pulverized, and milled for ten minutes utilising small-milling machine. WGP particulates with 25 µm in avg. particle-size have been employed as a reinforcing-particulate constituents that have contributed to the development of sustainable composites as illustrated in the Fig. 5. the chemical-constituents have been elucidated in Table 3. Three natural materials-limestone, soda ash, and silica sand-have been employed to make glass-bottles. The components for the glass-bottles are combined with recycled-glass, or “cullet.” Glass bottles’ main ingredient is “cullet”. The “cullet” is also mixed with trace amounts of “magnesium”, “Sulphur trioxide”, “barium oxide”, “ferric oxide”, and “aluminium oxide”. Colors in the bottles can be accomplished through the addition of several materials; including translucent white glass (through the use of incorporating phosphates, fluorides, Ca, ZnO, and tin), a yellowish-green colour (using chromium oxide), Green (using Cu, Cr, Fe), blue–greens (Co, Cr), Blue (cobalt oxide, Cu), Purples and reds (nickel oxide, Mn), Black (Cu, C, magnesia, Fe). Figure 4a,b has displayed the photograph of “waste glass”, and “waste glass powder”. The characteristics and for WGP has contributed to resistance to wear, the inclusion of WGP particulates has influenced by strengthening the fracture-toughness thereby enhancing the resistance to crack-initiation as well as crack-propagation. The utilisation of WGP as recycled materials has contributed to an economical viable way, and sustainable manner. WGP has been incorporated into construction materials such as concrete building materials to enhance their resilience, resistance to cracking or damage, toughness, long-lasting viability or reliability, durability and strength. WGP particulates have been employed in composites for a broad spectrum of applications, this material offers an ecologically viable and environmentally-sustainable replacement for conventional reinforcing-particulates^15^. In addition, WGP has been utilised in the development of insulating materials as a result of its thermal characteristics. Figure 6 has illustrated the XRD analysis for WGP. Powder XRD of waste glass has depicted the existence of SiO_2_, CaO, Na_2_O, and Al_2_O_3_ phases. Each phase, such as SiO_2_, CaO, Na_2_O, and Al_2_O_3_, will contribute different peaks in the diffraction pattern at particular angles (2*θ* values). Each crystalline phase can be recognized by these peaks, which act as fingerprints. It describes the percentage of the material that has changed from an initially amorphous (non-crystalline) state in the context of waste glass to crystalline phases. By identifying distinct diffraction peaks, powder XRD can identify the existence of crystalline phases. A phase that is immensely crystalline have sharp, intense peaks, whereas an amorphous phase has a broad, featureless pattern. Higher levels of crystallinity mean that more waste glass has crystallized into distinct phases like SiO_2_, CaO, Na_2_O, and Al_2_O_3_.

**Table 3:** Chemical-constituents of WGP particulates.

Compounds	SiO_2_	Na_2_O	CaO	Al_2_O_3_	Remaining compounds (Magnesia, Ferric-oxide, and Potassium-oxide)
Content (wt. %)	71.8	10.94	6.38	1.27	9.61

### Reinforcements: secondary reinforcement material

**Figure 7:**
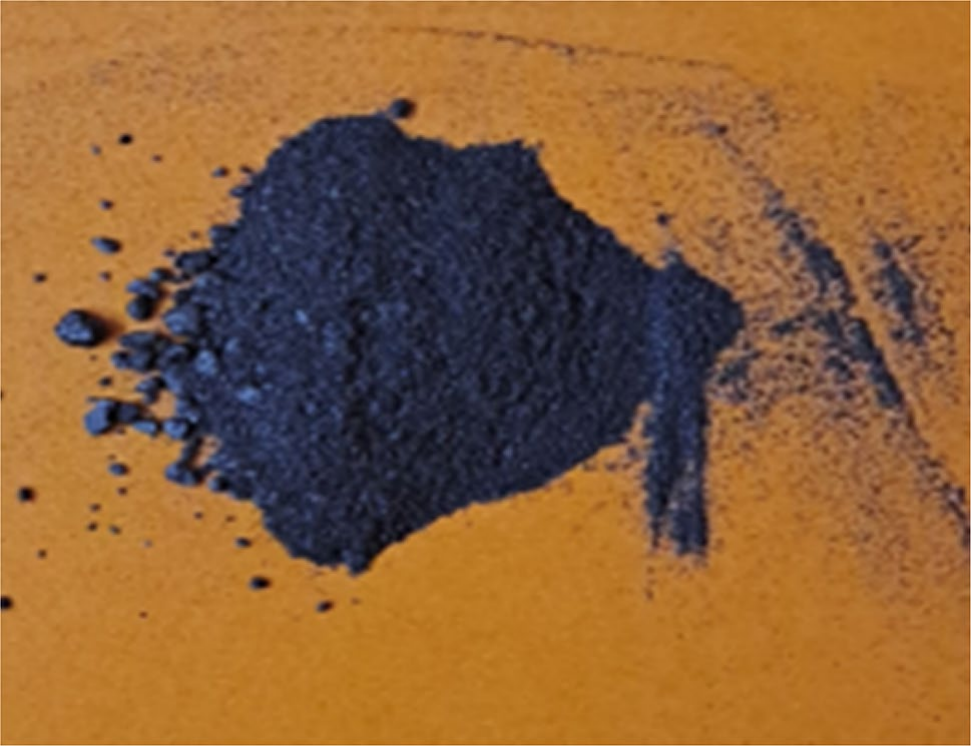
Illustration of silicon nitride (Si_3_N_4_) powder.

**Figure 8:**
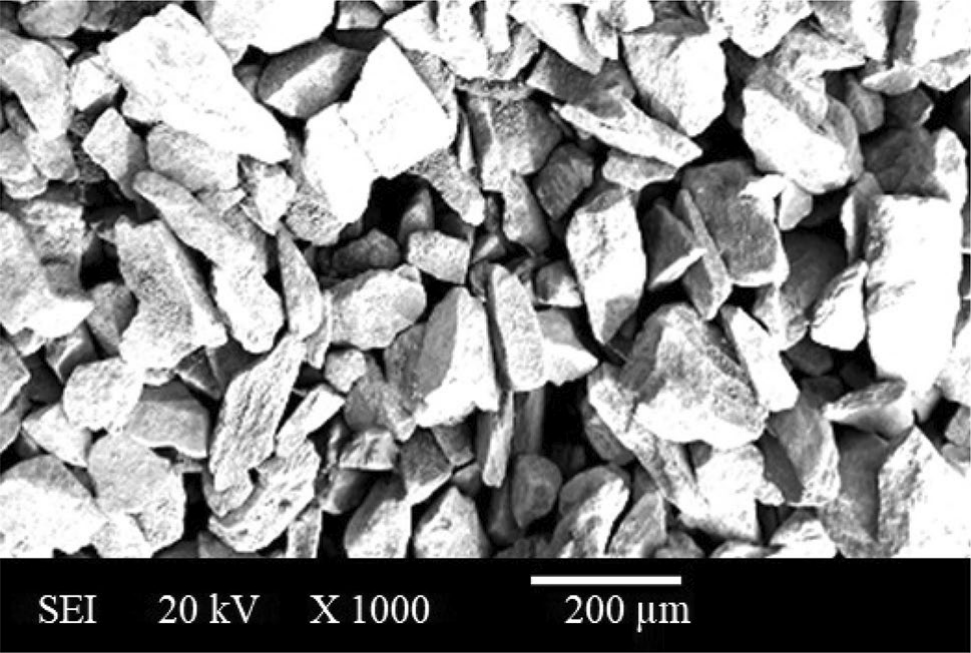
SEM image of Si_3_N_4_ powder.

**Figure 9:**
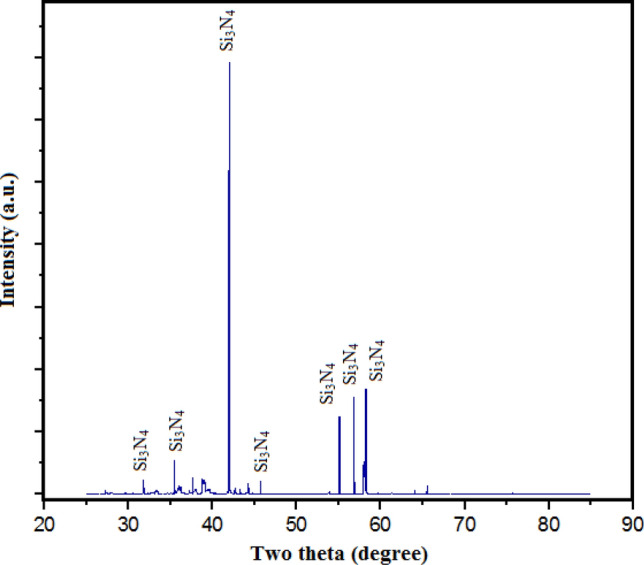
Powder XRD of Si_3_N_4_ powder.

Silicon Nitride (Si_3_N_4_) powder is a compound that has been constituted of the elements, silicon and nitrogen as depicted in the Fig. 7. Si_3_N_4_ with an avg. particle-size of 20 µm is a superior thermodynamically recognised material in powdered form as exhibited in the Fig. 8. Therefore, Si_3_N_4_ powder is the most demanding commercial powder. Si_3_N_4_ has facilitated or aided to a strengthening of mechanical characteristics and the enhancement of resistance to wear within the composite. In particle form, Si_3_N_4_ reinforcing-constituents have exhibited thermodynamic stability. Cutting tools have commonly utilised the Si_3_N_4_ as a result of its superior hardness as well as resistance to wear. Si_3_N_4_ reinforcing-constituents have been employed in ball bearings to mitigate wear, and strengthen long-lasting viability, reliability, performance functionality, and durability^16,17^. Si_3_N_4_ reinforcing-constituents have been utilised in high-temperature parts, including furnace-parts. A broader-spectrum for crystalline-phases for Si_3_N_4_ have been identified, including beta-Si_3_N_4_, alpha-Si_3_N_4_, and hexagonal Si_3_N_4_ (h-Si_3_N_4_). The XRD examination has illustrated that the resulting crystal’s lattice-structure is hexagonal in shape. Though, Si_3_N_4_ contamination by silicates and iron took place due to the use of low-purity raw silicon. Application of Si_3_N_4_ particulates can be observed in various fabrication parts such as Solar cells, integrated circuits, insulators, cutting tools, Medical devices, High-temperature material, Bearings, exhaust gas control valves, rocker arm pads, spark-ignition engines, turbochargers, glow-plugs, etc. Figure 7 shows the photograph of Si_3_N_4_ powder. Powder XRD of Si_3_N_4_ Powder shows 99% purity used in the present study (Fig. 9). Alpha-Si_3_N_4_, beta-Si_3_N_4_, and hexagonal Si_3_N_4_ (h-Si_3_N_4_) are some of the various crystalline phases of Si_3_N_4_. The specific phase(s) present in the sample can be determined by apparently observing for distinct diffraction peaks in the pattern at particular angles (2θ values). Due to each phase’s distinct crystal lattice structure, it has displayed a distinct pattern.

Amorphous or less crystalline Si_3_N_4_ phases have exhibited broad, diffuse peaks, while highly crystalline Si_3_N_4_ phases have displayed sharp, well-defined peaks. By contrasting peak intensities and shapes with reference patterns, it is possible to determine the level of crystallinity. A higher level of crystallinity implies that a greater proportion of the sample has undergone crystalline structure transformation. Additionally, it can be inferred from the XRD pattern that the hexagonal Si_3_N_4_ phase (h-Si_3_N_4_) has a distinctive hexagonal crystal lattice structure. It is possible that a sizeable portion of the Si_3_N_4_ powder has changed into crystalline phases because sharp, well-defined peaks suggest a high degree of crystallinity. Broad peaks might be an indication of amorphous or less crystalline regions. A deeper comprehension of the properties of the material is made possible by the determination of the crystal-structure, which offers intuitions into the arrangement of silicon and nitrogen atoms within the lattice of the identified Si_3_N_4_ phases.

### Development of composites using vacuum stir casting

**Figure 10:**
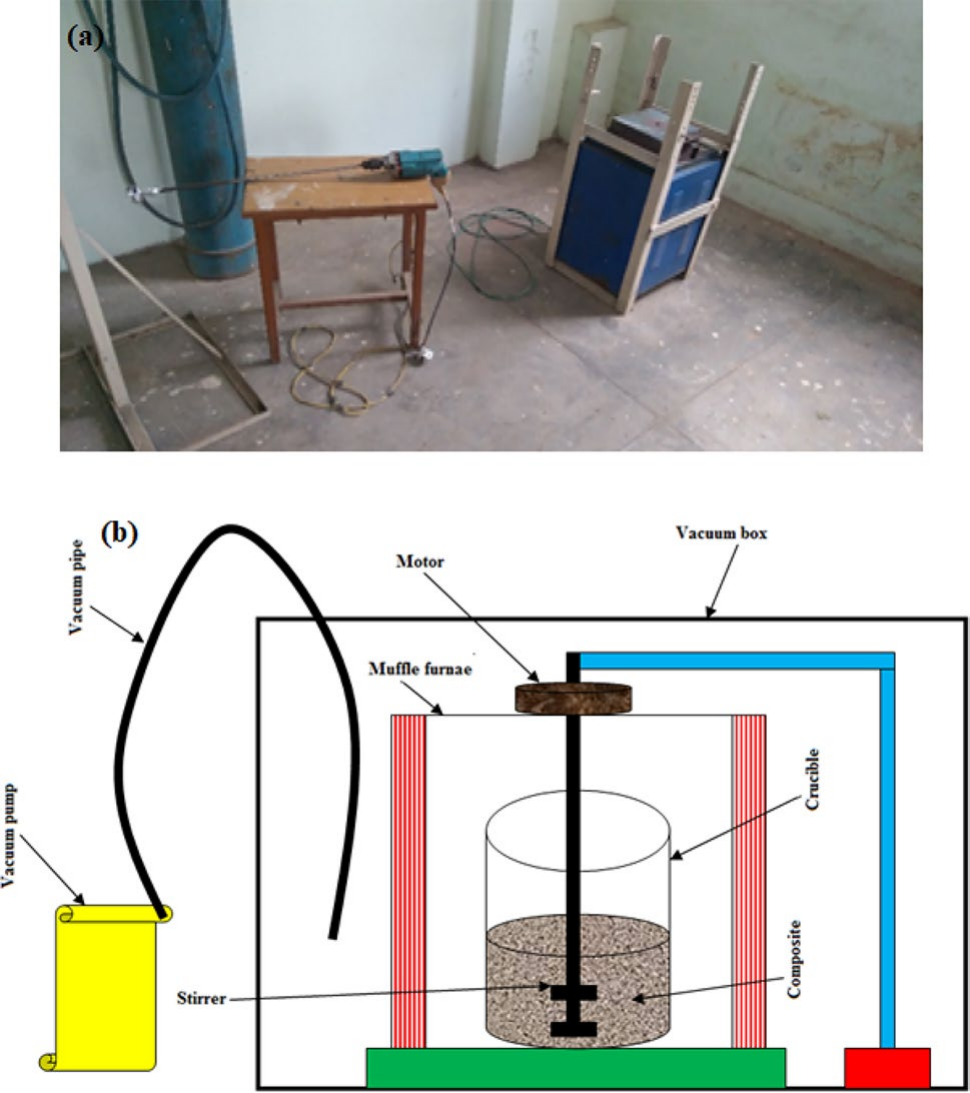
(**a**) Experimental set-up, (**b**) Schematic representation for the fabrication of Mg-MMCs.

**Figure 11:**
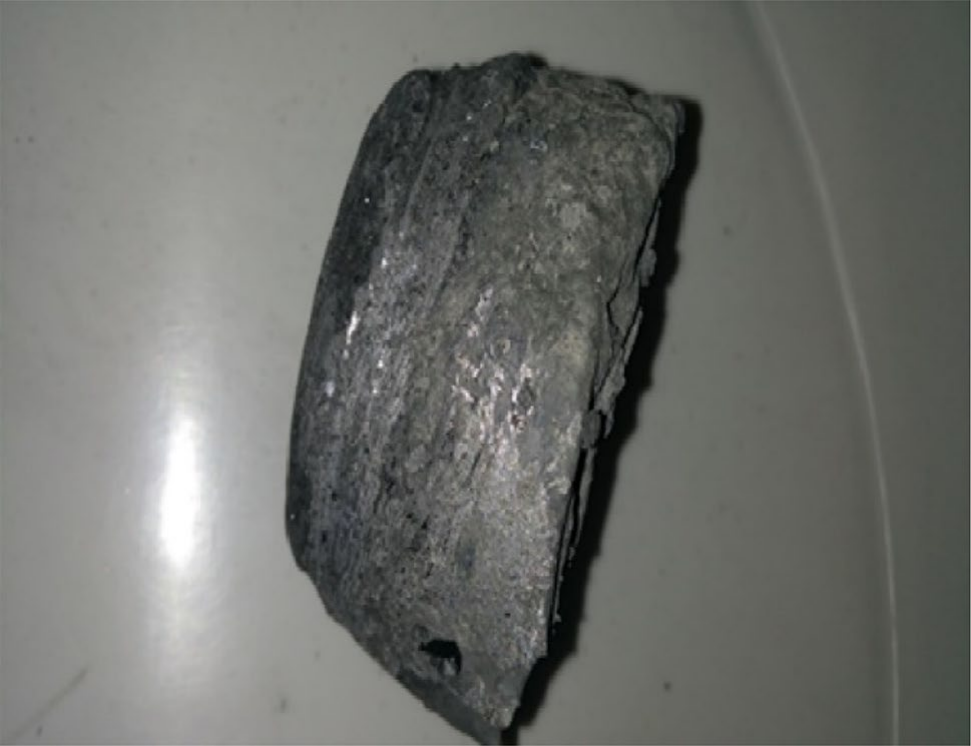
Photograph of developed Si_3_N_4_/WGP/AZ91D Mg-MMCs samples.

The composite was formed using an adaptation of the stir-casting procedure (Fig. 10a,b). The electric furnace heated the magnesium alloy to a temperature of around 680 °C. Additionally, while adding the reinforcement particle combinations to the molten matrix material, WGP and Si_3_N_4_ particulates have been heated. During the casting-method, the mould was 300 °C preheated. The parameter’s during processing has been depicted in Table 4. The “preheated reinforcing particulates” have been introduced to the matrix-material in accordance with the proportions stated in Table 5 once the magnesium alloy has completely melted (680 °C). The vertex was produced by employing a stirrer, while the AZ91D-matrix was further strengthened with pre-heated reinforcing-particulates utilising a hopper in compliance with the formulations as listed in Table 5. In a graphite-crucible, the AZ91D-matrix and reinforcing-particulates have been blended. The homogeneity or uniformity-dispersion of the blend-combination have been attained through the agitating process, which facilitated or allowed or aided an even-distribution for the reinforcing material within the AZ91D-matrix. In order to prevent the exposure of air’s contact to the molten magnesium alloy, a vacuum pump as well as a vacuum glass cover box were employed^18,19^. With a vacuum pump’s calibrated to approximately 50 mbar, a super-vacuum was developed within the vacuum glass cover-box. An agitator constructed of mild steel was employed in this research to agitate the composite material. A stainless-steel agitator has been utilised in order to avert any potential contamination. Uniformly consistent rate of stirring or speed of agitation and duration have been employed throughout the preparation of each sample. A 125 rpm of stirring-speed, and 5 min of stirring-duration have been maintained throughout the processing of every sample. The agitator has been removed once the composite has reached the mushy-stage condition. Additionally, a Mg-based composite blend-combination in the mushy-zone was being poured within vacuum environmental conditions to greyish cast-iron moulds. The casting-mould was being encased or enclosed in an enclosed or sealed vacuum-box. Additionally, Zircon/Water-based shield barrier coating-layers has been applied to the casting-mould with the aim to avoid the formation of pore-cracks, fractures, as well as ruptures inside the composite throughout the process of separation of casted specimen’s subsequent solidification. Following this, the resulting blend is then poured into an appropriate mould and then allowed to solidify. After the composite material has cooled down, it gets removed from the mould, allowed to air-dry, and then undergoes finishing processing operations in order to achieve the desired shape and dimensions as exhibited in the Fig. 11. In addition, various casting parameters have influenced the mechanical characteristics of the composite such as stirring-speed, stirring-time, wt% of reinforcement, pre-heat temperature of reinforcement, and stirrer material. To keep the molten Mg-alloy from coming in contact with the air, a vacuum-pump and vacuum-box were being employed as discussed in the former statements. In this investigation, the AZ91D-based composite was blended utilising stainless steel as a stirrer material. To develop every specimen, the stirring duration and speed were maintained consistent. For the growth of every respective fabricated composite specimen’s, the “stirring-speed”, and “duration” were maintained at 125 rpm and 5 min, respectively. The stirrer was taken out once the composite material had become mushy.

**Table 4:** Stir casting parameters for the developed AZ91D MMCs.

Parameters during casting process	Values
Stirrer material	Stainless steel
Blade-angle	40°
Blades-speed	125 rpm
Pre-heating chamber temperature	680 °C
Furnace-temperature	750 °C
Holding-duration	300 s
Shield furnace environment	Argon
Furnace	Electrical muffle-furnace

**Table 5:** Composition formulations of developed composites.

S. no	Compositions	Waste glass powder (wt%)	Si_3_N_4_ powder (wt%)	Codes for formulated composites
1	“AZ-91D + 1.5% WGP + 7.5% Si_3_N_4_”	1.5	7.5	A1
2	“AZ-91D + 3% WGP + 6% Si_3_N_4_”	3	6	A2
3	“AZ-91D + 4.5% WGP + 4.5% Si_3_N_4_”	4.5	4.5	A3
4	“AZ-91D + 6% WGP + 3% Si_3_N_4_”	6	3	A4
5	“AZ-91D + 7.5% WGP + 1.5% Si_3_N_4_”	7.5	1.5	A5
6	“AZ-91D + 0% WGP + 9% Si_3_N_4_”	0	9	A6
7	“AZ-91D + 9% WGP + 0% Si_3_N_4_”	9	0	A7

### Testing for the developed Si_3_N_4_/WGP/AZ91D Mg-MMCs composites

**Figure 12:**
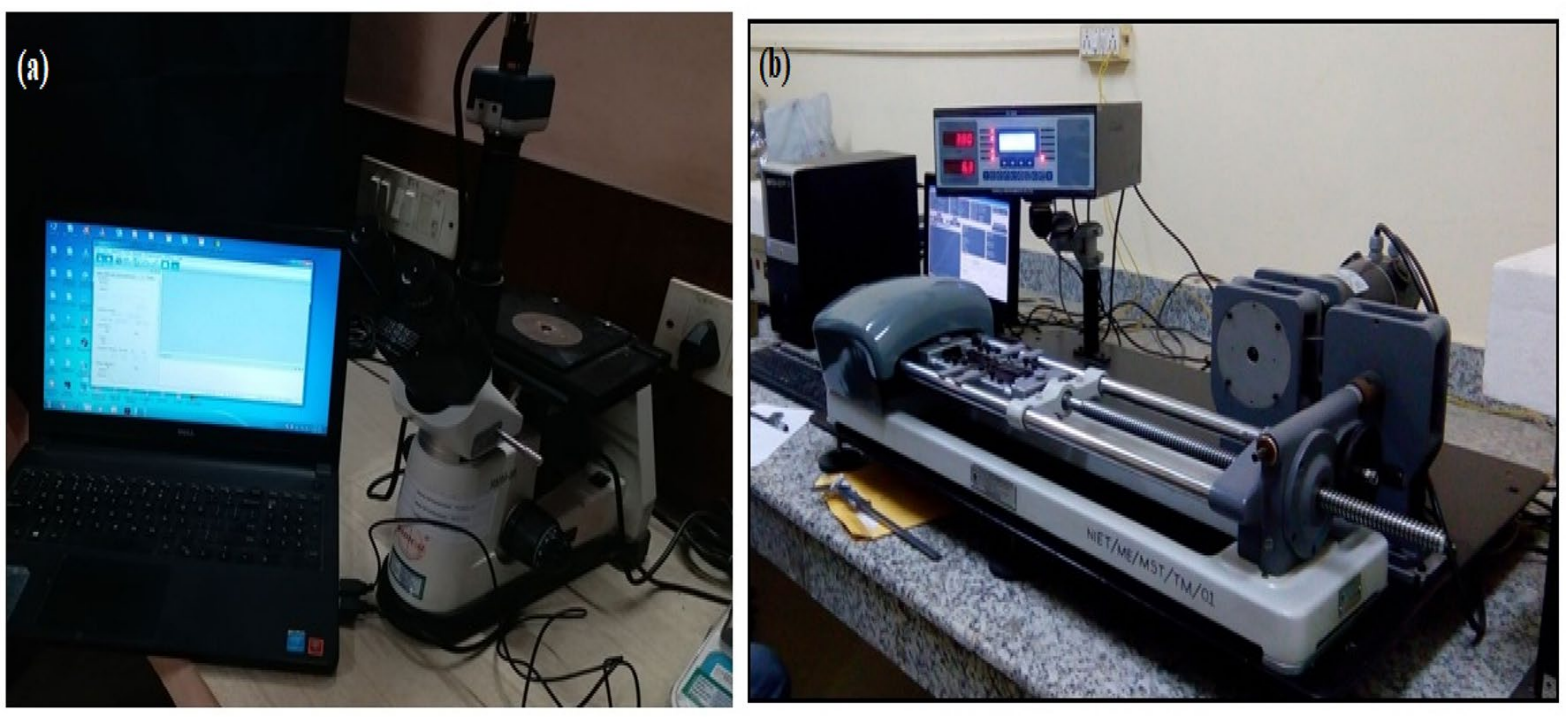
(**a**) Optical microscopic set-up, (**b**) Tensometer for tensile-testing.

The mechanical tests were conducted employing a Vickers-hardness testing for the measurement of hardness test and a Universal testing machine (UTM) for tensile testing. Calibration of UTM has been conducted to assure reliable, precise and accurate outcomes in mechanical examinations. The process entailed confirming, validating the functionality performance, and capability for the displacement-transducer, assuring that the load-cell as well as specimen have been positioned correctly, accurately oriented, properly aligned, and affirming the degree of precision of the forces measurement system. The tensile load is applied to a specimen clamped between two holders up until it fractures throughout the tensile examination by utilising the ASTM E8/E8M as exhibited in the Fig. 12a. The characteristics including the “ultimate tensile strength”, “yield strength”, and “elongation” have been assessed by this method of testing. The calibration method for the hardness-test apparatus was performed in a comparable manner. In order to properly calibrate a hardness measuring apparatus, its readings have been compared to a standard-reference and the machine was adjusted as required by utilising the ASTM-E18 standard. Samples for the fatigue-test have been prepared in compliance with the ASTM-E606 standard. Both optical microscopy as well as SEM analysis have been employed to examine the metallic characterization of the developed AZ91D Mg-MMCs as illustrated in the Fig. 12b. SEM analysis has been employed for higher-resolution imaging. In this study, “secondary electron” (SE), as well as “back-scattered electron” (BSE) detectors have been employed for compositional contrast as well as surface imaging, respectively. A motorized-stage is employed to attain accurate sample-orientation, placement, and positioning. To mitigate electron scattering, a vacuum-environment was being employed within the chamber. In contrast, micro structure test has entailed a microscopic examination of the material’s structure, facilitating the identification of imperfections as well as abnormalities or inconsistencies or irregularities that may have noticeable implications on its functional and operational characteristics. The samples of metallography have been developed in conformity with the ASTM-E3-95 standard. In order to evaluate the microstructure of the specimens, their surfaces were polished as well as etched with Keller’s reagent (10 ml HF + 50 ml H_2_O + 15 ml Hcl + 25 ml HNO_3_). This etchant is effective in revealing microstructural details and enhancing the contrast between different phases, allowing for a detailed examination of the sample’s surface morphology. SEM analysis has been employed for assessing the wear-pattern phenomenon, and worn-out surface characteristics for the composite samples with deteriorated surfaces. For the identification of phase-analysis, a “powdered X-ray diffractometer” was being employed. A mono-chromatic, higher-intensity X-ray source, like, “Cu Kα-radiation”. A sensitive, higher-resolution equipment, such as a “scintillation detector”. A “motorized-goniometer” is being utilised to ensure accurate sample positioning and orientation. A sample-holder is an apparatus designed for holding casted composite-specimens and facilitates rotation. The diffraction-patterns and outcomes reported below conclusively indicate the existence of reinforcement-particulates throughout the AZ91D-matrix alloy, even though in distinct or varying concentrations/proportions.

### Wear testing evaluation

Cylindrical-pins (AZ91D-based Mg-composites comprised of distinct formulated compositions of WGP and Si_3_N_4_ particulates) measuring 6 mm in diameter as well as 10 mm in height have been employed throughout the Pin on Disc investigation trial-testing employing ASTM-G65 standard. The diameter as well as thickness of the (235-HV10 hardness) cast-iron discs have been 63 mm as well as 6 mm, respectively. Among the disc and pin, a thick layer of solid-lubricants has been applied to simulate the actual realistic operational circumstances. The choice of pin on disc specifications has been determined by the findings of the pilot-test that was performed. The frictional, and wear characteristics of Mg-based composites comprising various blend-combinations of Si_3_N_4_ as well as WGP reinforcing-particulates have been reported at 5N loads, sliding-speed of 2 m/s, and a sliding-distance of 1000 m. In order to evaluate the tribological characteristics of the material, the coefficient of friction was being observed, measured, and recorded throughout the experiments. SEM analysis has been employed for assessing the wear-pattern phenomenon, and worn-out surface characteristics for the composite samples with deteriorated surfaces.

### Corrosion testing

**Figure 13:**
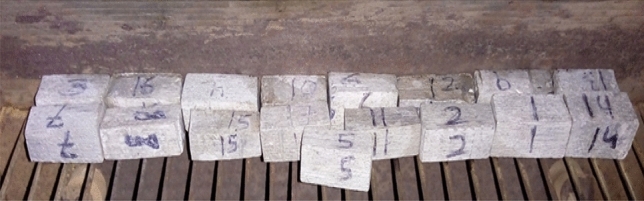
Samples for corrosion test.

The corrosion performance or activity of AZ91D-based Mg-composite samples with WGP and Si_3_N_4_ reinforcing-particulates involves immersion in a 3.5 wt% NaCl corrosive-media solution for 120 h as portrayed in the Fig. 13. The experimental method has included an assessing corrosion-resistance, measuring of weight-loss, and analyzing the surface-morphology. This method has aided in examining the protective efficacy of diverse multifaceted composites against corrosive environments. SEM analysis has been employed for assessing the corroded-surface phenomenon, pits, and corrosion-products for the composite samples.

## Rationale behind the selection of proposed material combination with wt. percent of the reinforcing-particulates for AZ91D + 1.5%WGP + 7.5%Si_3_N_4_ composites with scientific mechanism

From the in-depth literature scholarly analysis, the rationale motivation for examining the selected formulated composition of the proposed composites formulation have been elucidated based upon the following considerations^20–24^.

### Waste glass powder (WGP)

The analysis substantiated that the WGP particulates have been homogenously-dispersed or evenly-distributed across the AZ91D matrix, revealing that the blending process was efficient. The incorporation of 1.5% WGP by weight has strengthened the material’s mechanical characteristics considerably.

### Si_3_N_4_ (Silicon Nitride)

Si_3_N_4_ particulates have exhibited a comparable type or degree of a homogenously-dispersion within the AZ91D matrix as that of or likewise the WGP particulate. The incorporation of Si_3_N_4_ at a wt% of 7.5% has contributed to or yielded a substantial rise in resistance to wear.

### Synergistic combined implications for WGP and Si_3_N_4_ particulates

The findings for the research have reported that the incorporation of 1.5% WGP and 7.5% Si_3_N_4_ has culminated to a significant enhancement in resistance to wear, physicomechanical characteristics in accordance with the principles of sustainability. Also, the XRD analysis was employed for confirming the existence of harder phases within the AZ91D matrix, which were identified as the primary contributing factors for the reported enhancement of resistance to wear. Now, the analysis for the proposed developed composite material formulation, which incorporates Si_3_N_4_ and WGP as reinforcing agents in AZ91D magnesium-based composites, is based on a broader-array of factor considerations designed to strengthen the material’s mechanical as well as wear-resistant characteristics. The wt% of reinforcing-particulates selected are 7.5 percent for Si_3_N_4_ and 1.5 percent for WGP. Outlined below are the underlyiing principles with key fundamental concepts which underlay this composition as well as the possible upsides that it could offer:

### Microscopy analysis to ascertain homogeneous distribution

The optimum wt% formulated composition of 1.5% WGP and 7.5% Si_3_N_4_ reinforcing-particulates were evidently selected with the objective to accomplish an even homogeneous-distribution while retaining the AZ91D Mg-matrix’s integrity. The selected optimal wt% formulated composition for reinforcing enhanced interfacial-adhesion, and bond-strength, thus enhancing wettability characteristics. In accordance with the findings of microstructure analysis, WGP and Si_3_N_4_ particulates have been evenly dispersed throughout the AZ91D matrix, and wettability analysis have revealed the resilient interface-adhesion, durable interaction adherence, robust bonding-strength among the WGP as well as Si_3_N_4_ reinforcing-particulates and AZ91D-Mg matrix. Achieving this uniformity of distribution or even-dispersion has been accomplished by employing suitable techniques such as efficient melt-processing as well as agitation by the mechanical means. In addition, the underlying key objective is to attain an evenly homogeneous-distribution of WGP and Si_3_N_4_ across the AZ91D matrix. This has been affirmed by a microscopic analysis, which confirms that the reinforcing-particulates were dispersed uniformly throughout the AZ91D-matrix during the manufacturing technique. Ensuring this evenly uniformly distribution is of paramount significance in accomplishing strengthened material characteristics, as it promises a consistent homogenous-dispersion of reinforcements, which eventually makes a contribution to strengthened mechanical as well as wear characteristics.

### Enhancements of mechanical characteristics

In order to optimally utilise or maximise the reinforcement’s implications for mechanical characteristics, including hardness and tensile strength, the precise wt% of formulated composition of 1.5% WGP and 7.5% Si_3_N_4_ have been chosen. The incorporation of Si_3_N_4_ exhibited significant implications on the enhancement of mechanical efficiency, effectiveness, and performance functioning. The fatigue strength, ductility, tensile strength, and hardness of the developed composites have been evaluated. The findings confirm that the selected formulation, comprised of 1.5% WGP and 7.5% Si_3_N_4_, reveals strengthened mechanical characteristics. The material’s mechanical characteristics are considerably enhanced through the incorporation of 1.5% WGP and 7.5% Si_3_N_4_. The composite’s enhanced work-hardening as well as tensile strength seemed illustrative evidence of the beneficial constructive or favorable influence that the reinforcement had on the composite’s mechanical characteristics. Additionally, it has been reported that this particular combination of factors raises tensile strength, hardness, and fatigue strength, revealing that the wt% outlined above played a significant indispensable role in strengthening the mechanical characteristics of the composite.

### Enhancement of resistance to wear

The research emphasises on the enhancement of resistance to wear, and it has been unveiled that the synergistic combined-blend effect of 1.5% WGP and 7.5% Si_3_N_4_ yields a substantial improvement in this context. The wear-rate and coefficient of friction for the composite have been considerably reduced, in particular when the blend combination formulated composition of 1.5% WGP and 7.5% Si_3_N_4_ as reinforcing-particulate constituents was being utilised. Moreover, regarding applications where the material will be exposed both to friction and abrasive-wear, such as automotive or industrial components, this is of paramount significance. In addition, the strengthening of the wear-resistance is of crucial significance in applications that has subjected the developed composites to abrasive circumstances, as it effectively prolongs its functional service performance, longevity, durability, and operational lifespan.

### Structural analysis

The existence of harder ceramic refractory-phases within the AZ91D matrix, including α-Mg, Al_12_Mg_17_, SiO_2_, Si_3_N_4_, MgO, and CaO, can be identified through XRD analysis. Raised resistance to wear has been attributed to the existence of these phases. The selected wt% of reinforcements have facilitated/aided in the development of these harder ceramic refractory phases, which strengthen the composite against deformation, wear, degradation, deterioration and corrosion.

### Corrosion performance and mechanisms

The resistance to corrosion has been strengthened by the protective oxide shield layer or barrier on AZ91D Mg-matrix, and by the inclusion of silicon dioxide (SiO_2_) in WGP particulates. In addition, within a NaCl solution, the corrosion performance for the composites has been explored. The corrosion resistance of a material can be ascertained through weight loss measurements, while SEM images offer significant insights and valuable perspectives towards the development of corrosion-pits.

Hence, the choice for the proposed composite material composition—consisting of 7.5% Si_3_N_4_ and 1.5% WGP—is based on its capacity to aid in an evenly-dispersed, uniformly consistent distribution of reinforcing-particulates, enhanced physico-mechanical characteristics, strengthen resistance to wear, and reveal optimal desirable and promising performance during tribological and corrosion assessments. The extensive experimental as well as analytical methodology yields an in-depth analysis of the material’s functionality, performance effectiveness or capabilities, and ability to be utilised as a novel and enhanced substitution or replacement.

## Results and discussions

### Microstructure analysis and wettability analysis for the representative samples

**Figure 14:**
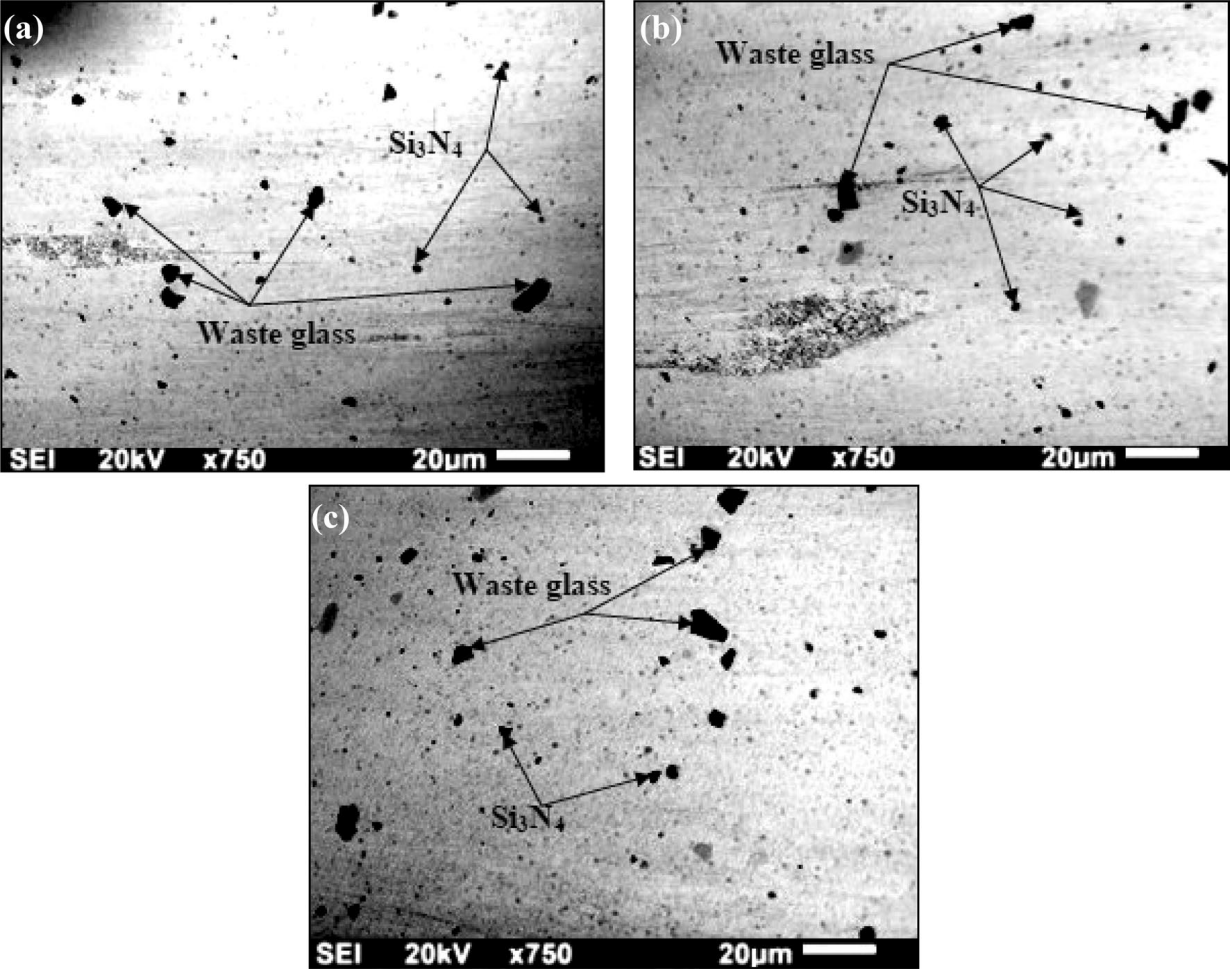
SEM micro-images of; (**a**) “AZ91D + 1.5% WGP + 7.5% Si_3_N_4_ composite sample”, (**b**) “AZ91D + 0% WGP + 9% Si_3_N_4_ composite sample”, (**c**) “AZ91D + 9% WGP + 0% Si_3_N_4_ composite sample”.

The developed AZ91D-based composites reinforced by employing WGP, and Si_3_N_4_ has been depicted in Fig. 14a–c using SEM images. The nearly consistent-separation of the WGP, and Si_3_N_4_ in AZ91D + 1.5%WGP + 7.5% Si_3_N_4_ composites is visible in the microstructures. For all compositions, equitable distribution can be seen though.

For composition A1, within the AZ91D matrix, the SEM images of this composition has revealed a nearly persistent division of waste-glass powder and Si_3_N_4_. The WGP and Si_3_N_4_ particles can be observe to be evenly distributed throughout the matrix owing to the analysis of its microstructure. This suggests that these additives are distributed within the AZ91D alloy fairly uniformly^20,21^.

The fabrication process’s appropriate mixing, the additives’ adequate distribution in the alloy melt, and perhaps interactions among the additives and the AZ91D matrix that stimulate even distribution are all factors that can be linked to the mechanism causing this distribution.

For composition A6, the SEM images has revealed a microstructure primarily constituted of the Si_3_N_4_ additive. Despite the absence of WGP particulate, it is anticipated that the Si_3_N_4_ particles will be distributed throughout the AZ91D matrix, possibly resulting in a distinct morphology from the original composition^22,23^. Si_3_N_4_ distribution may be influenced by manufacturing-related variables like the melting and mixing conditions.

For composition A7, SEM images of this composition has unveiled a microstructure primarily composed up of the WGP particulate within the AZ91D matrix. The powder particles of waste glass are probably scattered throughout the matrix. Once more, factors like melting and blending during fabrication could have an impact on the distribution.

Additionally, the fair-distribution of additives across all compositions, as observe in the SEM images, implies that the fabrication process was effective in achieving a comparatively homogenously-distribution of the WGP and Si_3_N_4_ within the AZ91D matrix^24^. This equitable distribution is influenced by a number of factors:

1. Employing appropriate methods for combining the additives with the AZ91D alloy, such as mechanical stirring, can aid assurance an additional even dispersion.
2. Effective melt processing has facilitated the uniformly-dispersal of additives within the molten AZ91D alloy by controlling temperature and stirring at the appropriate rate.
3. The AZ91D matrix and the additives may interact chemically or physically, making it easier for the additives to be distributed evenly.
4. Under controlled conditions, the additives can be kept from separating and settlement during cooling.
5. Meticulous assortment of Si_3_N_4_ and WGP particle sizes can influence how they disperse within the matrix.

Now, the Optical microscopic image (Fig. 16a,b) for AZ91D + 1.5%WGP + 7.5% Si_3_N_4_ composites have presented defect-free developed composite after solidification. The α-Mg grains shown by the red background can be detected in optical microscopic images for both “AZ91D + 1.5%WGP + 7.5%Si_3_N_4_”, and “AZ91D + 0%WGP + 9%Si_3_N_4_” fabricated formulated composites. The basal grains own larger sizes and with a smaller spherical second phase.

For composition A1, the absence of defects in the developed composites after solidification indicates that the additives were processed and distributed successfully in this composition. This observation is a result of several mechanisms, firstly, before the AZ91D alloy solidifies, Si_3_N_4_ and WGP particulates should be thoroughly mixed with it to ensure that the additives are distributed evenly throughout the molten metal. Secondly, the effective wetting among the additives and the molten AZ91D encourages uniform distribution and reduces the formation of flaws or voids. Thirdly, controlled solidification factors, including, cooling rate and thermal gradients, aid in preventing segregation and enable the composite to solidify without significant flaws. Fourthly, the effective incorporation of WGP and Si_3_N_4_ without introducing defects can be facilitated by meticulous selection of the particle sizes.

For composition A6, the absence of WGP particulate in this composition indicates that Si_3_N_4_ particles within the AZ91D matrix will have a foremost primary microstructure. The absence of WGP is the primary difference between these mechanisms and those considered in the prior composition.

In addition, the grains containing α-magnesium has been identified. Due to their distinctive characteristics, the primary phase of the AZ91D magnesium alloy, the α-Mg grains, can be identified in optical microscopic images. Differences in contrast make it simple to distinguish α-Mg grains in the optical microscope from the surrounding phases. Depending on the orientation of the microscope and the grain structure, the boundaries between α-Mg grains can appear as lines or curves. With the adequate etching methods, it is possible to increase the contrast among α-Mg grains and other phases, making it easier to discover these phases^25,26^.

Furthermore, the larger and lesser spherically symmetrical second-phase of basal grains have been reported. The basal grains (α-Mg grains with a basal plane) in these compositions have greater sizes when compared to a smaller spherical second phase (possibly Si_3_N_4_ or another phase), according to optical microscopy^27^. Crystallography and solidification mechanisms can be employed to elucidate the former statement. Firstly, the growth of basal planes is encouraged by the crystal structure of α-Mg, according to crystallography. As a result, the larger grains are inclined toward the basal-grains. Secondly, the second phase, which is moreover compact and spherical, might nucleate and grow differently than the base grains, giving rise to differences in size and morphology. Finally, during solidification environmental circumstances, the cooling rates and thermal gradients can affect the rates at which different phases grow, resulting in changes in grain size^28–30^. To increase characteristics, reinforcing particles (such as Si_3_N_4_ and WGP particulate) must be properly wettable. For the composition, “AZ91D + 1.5% WGP + 7.5% Si_3_N_4_ composites”, the findings of the current study demonstrated good-affinity of WGP, and Si_3_N_4_ granules with magnesium (Fig. 17a,b). The mechanical behaviour of composite materials is enhanced by adequate wettability. Interfacial adhesion determines wettability. The interfacial adhesion among the AZ91D and reinforcement particles is depicted in Fig. 17a,b. With AZ91D, WGP and Si_3_N_4_ particles developed strong bonds, as seen by this interfacial attraction. As wettability is determined by the equilibrium of force among cohesive and adhesive intermolecular contacts, it is the propensity of “reinforcing-constituent particulates” to retain adequate interaction with the surface of matrix-material. Wetting behaviour in metal matrix composites is influenced by a number of variables, including the “density” of the “matrix”, and “reinforcing-constituent particulates”, “temperature”, and “pressure”, “time of interaction”, “crystallinity”, “pathogens”, the “surface roughness” of the “reinforcing-constituent particulates”, “shape of the reinforcing-constituent particulates”, and others. The formation of adequate wettability in the matrix and reinforcement particles, nonetheless, is greatly influenced by the temperature of the reinforcement preheating process. The desorption of moisture/gases, pollutants or decrease of surface impurities, enhanced particle retention, and changed particle surfaces could all result from proper reinforcement preheating^31,32^. Si_3_N_4_ addition has a substantial influence on the interfacial layer between Si_3_N_4_ and aluminum. One of the main reasons for this is the formation of a reactive Si–Al–O–N layer at the interface, which is formed due to the reaction between the Si_3_N_4_ and the aluminum. This layer is crucial to the adhesion between the two materials, and its formation is dependent on the amount of Si_3_N_4_ added. Present study has shown that as the amount of Si_3_N_4_ added increases, the thickness of the interfacial layer also increases. Moreover, the “thickness of the layer” is directly proportional to the amount of “Si–Al–O–N phase” formed. This suggests that the Si_3_N_4_ addition promotes the formation of the Si–Al–O–N phase, which is responsible for improving the adhesion between the two materials. Furthermore, the Si_3_N_4_ addition also affects the morphology of the “interfacial-layer”. In the absence of Si_3_N_4_, the “interfacial-layer” tends to be discontinuous and irregular. However, the addition of Si_3_N_4_ promotes the formation of a smoother and denser layer, which contributes to improved adhesion between the materials^33^.

Moreover, as unveiled from the Fig. 17a–b, the several mechanisms can be employed to elucidate the considerable attraction of Si_3_N_4_ and WGP particulate to the magnesium-matrix (AZ91D) in the composite. Firstly, when the additives have a fierce-bonding for the magnesium-matrix, which promotes close contact at the interface, there is appropriate wetting. Surface energies affect wettability because they control how easily a liquid (melted magnesium) spreads over a solid surface (additives). Secondly, the chemical interactions can result in the formation of chemical compounds or robust bonds at the interface among the components of WGP, Si_3_N_4_, and magnesium. For instance, magnesium may react with the oxides in the WGP particulate to form stable compounds. Thirdly during solidification, the melt surrounding the additives may solidify and mechanically fix the particles in spot, escalating their interfacial attraction^33,34^.

Fourthly, the temperature and processing time both contribute to providing enough time for interfacial reactions to take place and for mechanical bonding to form.

Additionally, the interfacial attraction observed in the SEM images indicates that the WGP and Si_3_N_4_ particles have a fierce-bonding with the AZ91D matrix. Firstly, the mechanical interlocking, which results in increased bond strength, may be developed as the magnesium matrix solidifies around the additives. Secondly, at the interface, atoms from the additives and the matrix may diffuse, resulting in a diffusion zone with better bonding. Thirdly, the presence of reaction layers at an interface may be an indication of chemical bonding or the synthesis of intermetallic compounds, both of which can strengthen a bond^34,35^. Fourthly, an efficient stress transfer among the additives and the matrix is made possible by a strong interface, which improves load-bearing capacity. An analysis of the Microstructural-morphology of the fabricated AZ91D/WGP/Si_3_N_4_ composites have been enumerated. In context with the grain formation is concerned, as evidenced by the micro structures for “AZ91D/WGP/Si_3_N_4_ composites” is that the reinforcement’s weight-percentages have been evenly-distributed or uniformly-dispersed throughout the AZ91D-matrix. The shape, size, arrangement, as well as dispersion of these grains, that can be evident utilising optical microscopy, have had profound implications on the material’s characteristics. An even dispersion of reinforcing-particulates (WGP and Si_3_N_4_) appeared under the microscope within the AZ91D-matrix, revealing that grain-formation in the composite seemed efficient. In context with the grain structure is concerned, the structure of the grain for a material pertains to the arrangements, structure, and properties of its constituent grains. The optical microscopy images for the “AZ91D-Mg cast-alloy” offer insight into the grain-structure of magnesium (α-Mg). Differences in size of grains, and shape could potentially reveal distinct the process of solidifying circumstances or subsequent processing or post processing techniques. By employing optical microscopic pics, the alpha-Mg grain or particulate structure has been examined. Indicators are the boundaries that separate individual granules, while discrepancies in size and shape may indicate distinct solidification conditions or post-processing techniques. In accordance with the grain refinement is concerned, process of refinement of grains has been accomplished through the inclusion of reinforcing-particulates including WGP and Si_3_N_4_. The incorporation of Si_3_N_4_ into the AZ91D-based composites have been reported to substantially raise its mechanical characteristics as well as resistance to wear, demonstrating that it could be contributing to the refinement of grains throughout the AZ91D-MMCs. The microstructural examination revealed that these particulates are evenly-dispersed or uniformly across the AZ91D-matrix, which enhances the material’s mechanical characteristics. In context with the phenomenon and mechanisms described for micro structure examination is concerned, a wide spectrum of parameters, including blending techniques, melting processing, interactions between chemicals, and precisely predetermined controlled solidifying circumstances have influenced the microstructure of a material. The SEM images provide insights into the additives’ dispersion, while the efficient optimised manufacturing method assures evenly-distribution. The microstructure reveals that the additives are even uniformly-distributed, thus offering substantiation for the manufacturing process’s efficiency. This evenly distributed dispersion has been facilitated by mechanisms including suitable blending-techniques, efficient melting-processing, and prospective chemical-interactions.

In accordance with the enhancement of characteristics as a consequence of the grain-fineness process is concerned, the mechanical characteristics, such as hardness, resistance to wear, and tensile strength, are substantially enhanced through the incorporation of WGP and Si_3_N_4_. The phases formed have been examined through microstructural analysis, which comprises of XRD, and SEM analyses. This insight contributes to the enhancement of characteristics. As illustrated by SEM images, the micro structure of the composite has been strengthened as well as uniformly homogenised or dispersed by means of the utilisation of a vacuum-pump. The Halle-Petch connection indicates that a reduction in the size of the grains conduces to a rise in the yield strength of a composite, thus signifying the favourable implications of the refinement of grains on the composite’s mechanical characteristics.

In addition, the differentiation among the two reinforcing-particulates (WGP and Si_3_N_4_) can be identified through an analysis of the subsequent accompanying findings, as for the sample with AZ91D + 1.5%WGP + 7.5% Si_3_N_4_ composite is concerned, the morphology for AZ91D matrix waste glass particles and Si_3_N_4_ exhibits a nearly uniformly or evenly-dispersal and distributed structure. The SEM images indicate that the WGP, and Si_3_N_4_ particulates have been homogenously-dispersed, confirming an efficient blending as well as distribution within the AZ91D-matrix. For sample with AZ91D + 0%WGP + 9% Si_3_N_4_ composite is concerned, the predominant presence for Si_3_N_4_ in the microstructure signifies that the composition is primarily composed of Si_3_N_4_ additive. The Si_3_N_4_ particulates were apparently dispersed across the AZ91D-matrix. A dispersion in which processing factors including melting and blending circumstances exert an influence. It has been believed that Si_3_N_4_ particulates will eventually get dispersed across the AZ91D-matrix, affecting the structure’s microstructure and morphology in a manner that is different from composition AZ91D + 1.5%WGP + 7.5% Si_3_N_4_ composite, regardless of the absence of WGP. For sample with AZ91D + 9%WGP + 0% Si_3_N_4_ composite is concerned, the micro structure for the AZ91D-matrix comprises primarily of WGP, with particulates apparently dispersed throughout the matrix. The dispersion of WGP particulates throughout the AZ91D-matrix can be impacted by parameters including melting as well as mixing that take place during the manufacturing process.

WGP particulates have been dispersed throughout the AZ91D-matrix.The dispersion has been impacted by factors of manufacturing. The variables which impact dispersion have been elucidated as^36,37^, firstly, the adequate processes for blending additives with AZ91D-matrix has aided a homogeneously-distribution. Then, efficient melting manufacturing controls agitating-speed or stirring-rate, and temperature thus ensuring a homogeneous-distribution. Then, the evenly-dispersion is facilitated by chemical or physical interactions that occur among the AZ91D-matrix as well as additives. Whereas cooling, separation and settlement are prevented by controlled circumstances. Meticulously choice of sizes of particulates including, Si_3_N_4_ and WGP has an influence on how they distribute within the AZ91D-matrix.

In general, the SEM images demonstrate that the manufacturing technique effectively incorporated WGP as well as Si_3_N_4_ particulates into the AZ91D-matrix in a comparatively uniformly-dispersion. Considerations like suitable blending techniques, efficient melting-processing, probable physical or chemical interactions among the AZ91D-matrix and additives, as well as controlled circumstances throughout processing have all implications for the degree of uniformity or homogeneity of the distribution.

The examination of the microstructure for the composites, as illustrated in Figs. 14a–c, 16a,b, and 17a,b reveals significant characteristics. The systematic investigation of the microstructure has comprised an extensive-array of mechanisms as well as phenomena’s. Firstly, the performance effectiveness blending and dispersion, by employing appropriate blending techniques and circumstances, WGP, and Si_3_N_4_ are uniformly-dispersed throughout the AZ91D-matrix. An optimal pre-heating of reinforcing particulates facilitates proper wettability, which in turn enhances intermolecular-interactions between molecules and bonding adhesion-strength^38^. The AZ91D-matrix and additive interactions lead to the reported uniformity of distribution in SEM images. Secondly, the solidification devoid of imperfections or defects or irregularities, as exhibited from the Fig. 16a,b, the Optical microscopy pics for composites that comprised of AZ91D + 1.5%WGP + 7.5%Si_3_N_4_ has revealed solidification without any flaws or imperfections. The exemplary performance, efficient functioning, and dispersal of additives throughout this composition are considerations which are responsible for the absence of imperfections. Thirdly, characteristics of the microstructure, as exhibited from the Fig. 16a,b, the α-Mg grain structure is discernible through optical microscopy, exhibiting variations in shape as well as size. Composition A1 has revealed an efficient wettability, controlled-solidification, and precise control of size of particles, as exemplified by an absence of imperfections. Fourthly, the physical and chemical interactions, for an additional evenly homogeneous-distribution, the chemical reactions or physical interactions among reinforcing-particulates, and the AZ91D-matrix have been accountable for it. An optimal efficient stress-transfer or distribution of stress among additives as well as the matrix contributes to a rise in load-bearing performance. Finally, the implications of process operating circumstances, in order to attain uniform dispersion, efficient melting-processing, controlled-temperature, and agitation rates or stirring-speed during manufacturing are extremely significant. Parameters including agitating speed, agitating duration, and wt% proportion formulations of reinforcing-additives exert a profound impact on the morphology.

**Figure 15:**
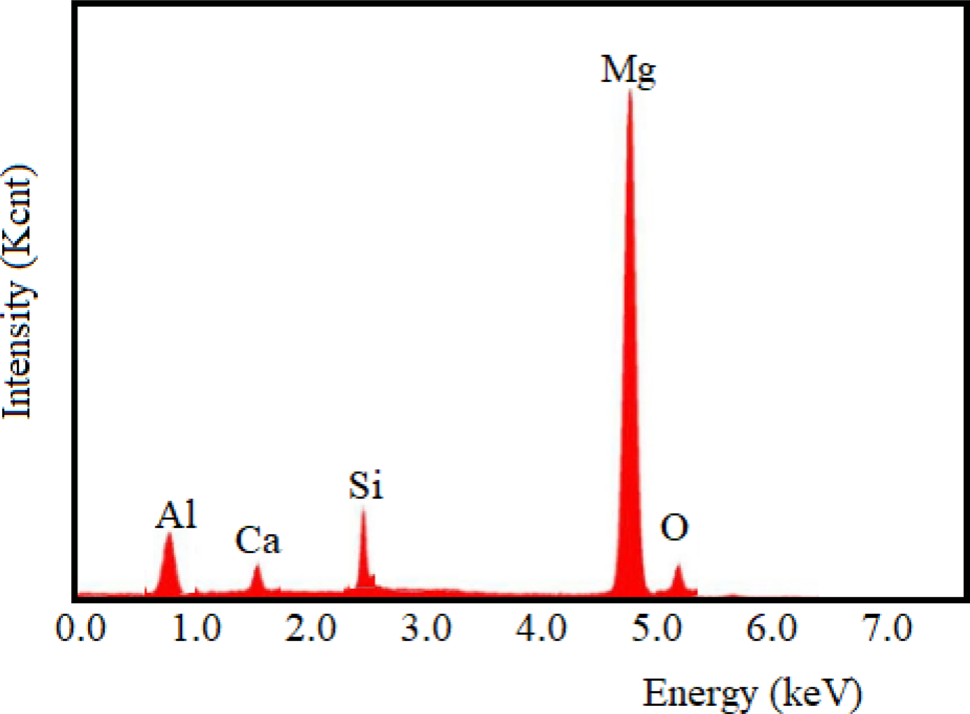
Representative EDS pattern of “AZ91D + 1.5% WGP + 7.5% Si_3_N_4_ composite sample.

Moreover, the EDS outcomes have been employed to discern the particular phases that have been generated at the interfacial-layer contact among the AZ91D-matrix and Si_3_N_4_ and WGP reinforcing-particulates. In this case, as exhibited from Fig. 15, the apparent existence of the respective magnesium peak, silicon peak, aluminium peak, calcium peak, and oxygen peak in the EDS-spectra analysis has revealed conclusive evidence for the inclusion of the corresponding elements throughout the 1.5%WGP/7.5%Si_3_N_4_/AZ91D Mg-matrix composites. The occurrence of this most noticeable significant or highest-peak with a spectrum indicates that the magnesium was the primarily predominant element with a particularly higher-concentration, despite various other elements have additionally been detected in varying amounts or proportions. This substantiates the assertion regarding the development of resilient, hard, stable, durable, high strength, tough, corrosion-resistant, resistance against deformation, and robust protective barrier layer composites^36–38^. Moreover, the reinforcing-constituents utilised in this investigation is composed of the Si_3_N_4_, and WGP particulates. The EDS findings would provide confirmation that these elements (Si, Ca, Al) have been presented in the developed vacuum stir-casted AZ91D composites. In addition, a uniformly-dispersion, and homogeneity arrangement of Mg, Al, Si, and Ca, elements have indicated the presence of an interfacial-layer among the AZ91D-matrix and Si_3_N_4_ along with WGP reinforcing-particulates that is well bonded, homogenous, and evenly-dispersed or arranged in consistency.

**Figure 16:**
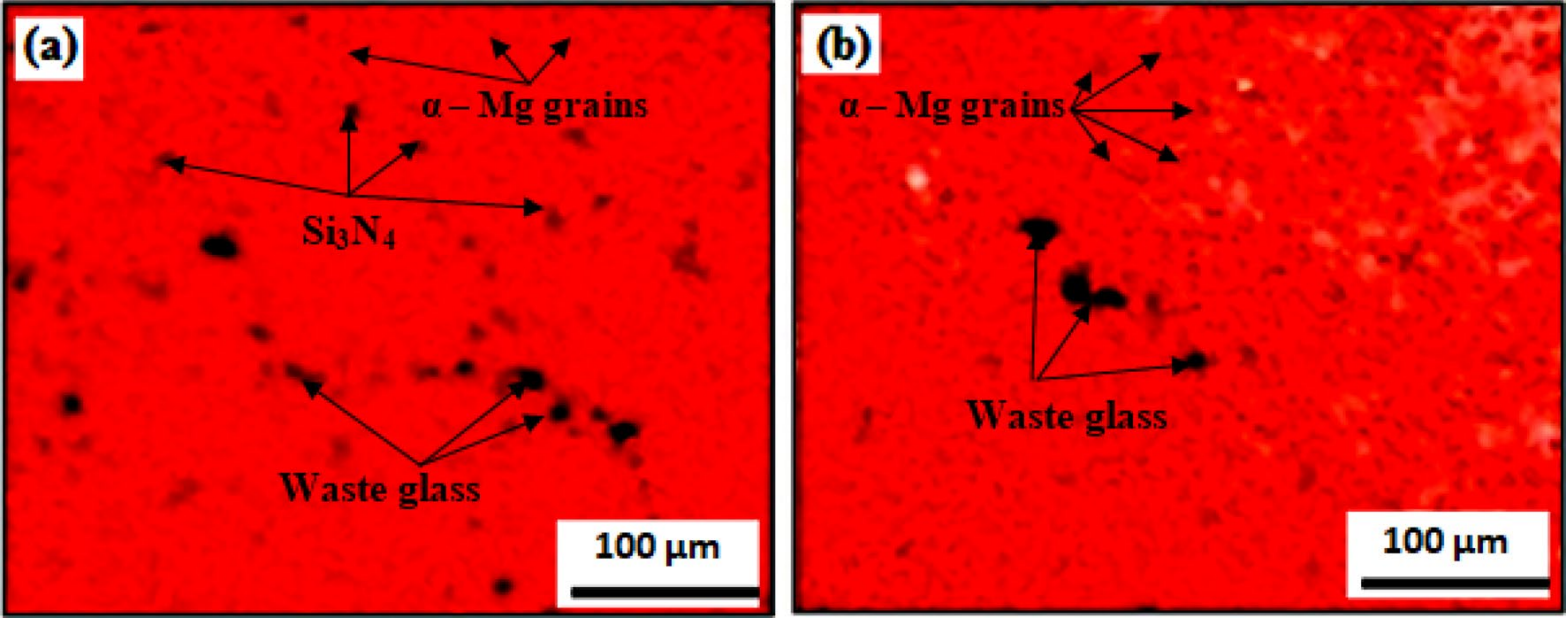
Optical-microscopic images of; (**a**) “AZ91D + 1.5% WGP + 7.5% Si_3_N_4_ composite sample”, (**b**) “AZ91D + 0% WGP + 9% Si_3_N_4_ composite sample”.

**Figure 17:**
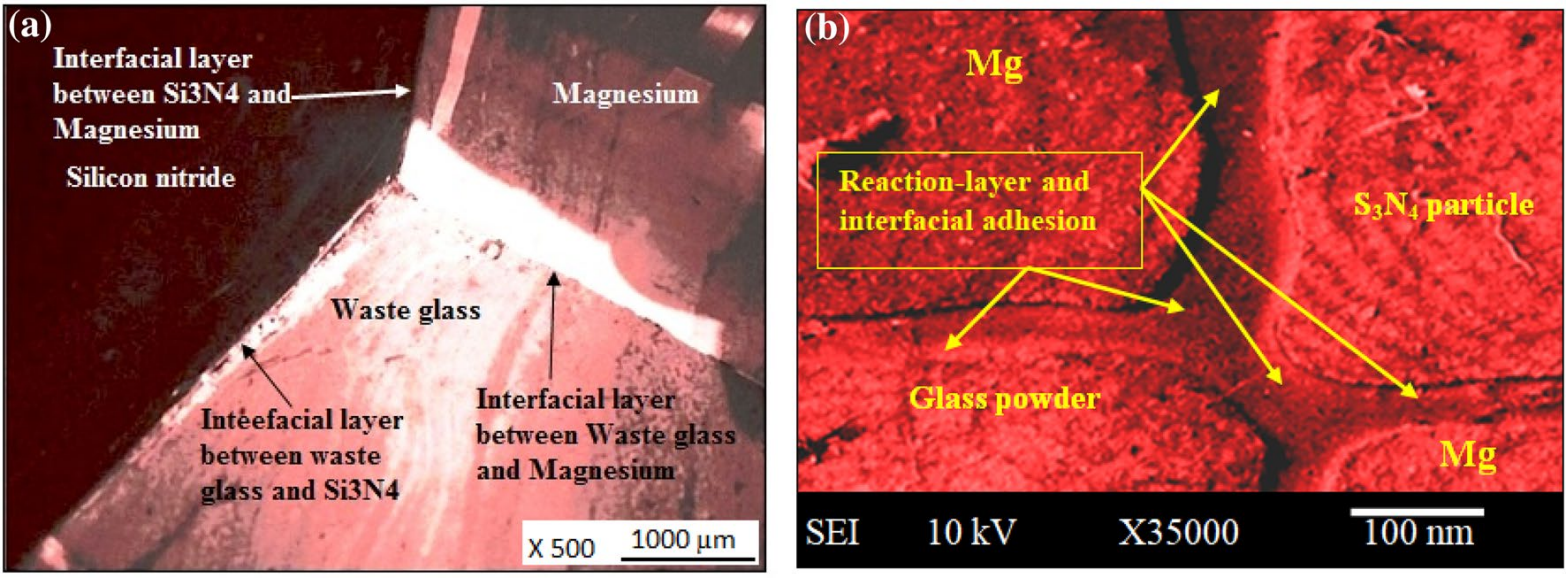
(**a**) Reaction-layer of “AZ91D + 1.5%WGP + 7.5% Si_3_N_4_ composites”, (**b**) Reaction-layer and interfacial adhesion of AZ91D + 1.5%WGP + 7.5%Si_3_N_4_ composites.

The wettability examination, micro structure, and optical examinations for the fabricated composites are displayed in Figs. 14a–c, 16a,b, and Fig. 17a,b. The methodical examination involves exploring the underlying mechanisms that are accountable for the observable micro structural characteristics. The resistance to wear is enhanced caused by the evenly distributed distribution of WGP and Si_3_N_4_ reinforcing-particulates as shown in the microstructure (Fig. 14a–c). The appearance for defect-free composites in the SEM images (Fig. 17a–b) following the process of solidification indicates that the additives have been effectively dispersed with efficient processing manufacturing method^38,39^.

The optical microscopy analysis of the sample “AZ91D + 1.5%WGP + 7.5%Si_3_N_4_” reveals enhanced characteristics, as demonstrated by the larger α-Mg grain particulates and the reduced dimensions of the sphere-shaped-like second-phase (Fig. 16a,b). The investigation of wettability (Fig. 17a,b) has confirmed that AZ91D-matrix and WGP/Si_3_N_4_ reinforcing-particulates have developed strong adhesion bonding-strength, robust resilient-stability, and durability, strength, resulting in strengthening the composite’s mechanical characteristics. Parameters such as blending-techniques, melting-processing, and chemical/physical interactions could be attributable for these findings^39,40^.

In the broader scope of the existing research, the wettability study (Fig. 17a,b) and its correlation with interfacial-adhesion and bonding-strength between the surfaces have been examined with particular emphasis on the crucial function of a robust interface in order to strengthen load-bearing performance. The rationale incorporates parameters that impact wettability, including temperature, pre-heating for reinforcement, and the development of reacting-layers at interface-surfaces^40^. This is in accordance with significant academic studies concerning MMC’s.

An appropriate distribution of reinforcing-particulates, efficient wettability among additives as well as matrix, and robust interface-adhesion or bonding-strength between surfaces, are considerations that, in accordance with the relevant scientific literature, are contributing to enhanced mechanical characteristics and resistance to wear. The identification of harder phases through XRD analysis, including α-Mg, Si_3_N_4_, and other similar phases, seems to correspond with the existing research that reveals these phases aid to enhanced resistance to wear.

Furthermore, the implications of Si_3_N_4_ on interfacial-surfaces layer interaction and the development of Si–Al–O–N phases adheres to scholarly studies that emphasise the vitality of bonding between layers or bonding-strength between surfaces in composites. The implications of WGP reported impacts on fracture toughness, cracking initiation, and propagation correspond with the existing research that explores the impacts of reinforcing characteristics on the performance or behavioural characteristics of composites.

Now, in accordance with the phenomenon and mechanism of grain-fineness is concerned, the enhancement of characteristics is primarily attributable to the grain-refinement mechanism, the even distribution of reinforcing-particulates, interface-interaction, and the development of harder-phases^40–42^.

In context with the Composite Processing is concerned, the utilisation of the vacuum stir-casting process during composite manufacturing has thereby prevented oxidation, or degradation, or deterioration by ensuring the appropriate or suitable distribution of WGP and Si_3_N_4_ reinforcing-particulates. Now, in accordance with the homogenous-distribution is concerned, the microscopic examination corroborated that WGP and Si_3_N_4_ were distributed evenly throughout the AZ91D matrix. The grain fineness mechanism primarily depends significantly on this distribution since it promises a consistent homogenous-dispersion of reinforcing-particulates across the composite. Now, in reference with the micro structure enhancement is concerned, the examination of the microstructure evidenced an evenly uniform separation of WGP and Si_3_N_4_, revealing that the manufacturing technique (vacuum stir-casting) adequately achieved homogeneity or uniformity in the composite. Developing homogeneity is a critical indispensable and imperative aspect in the method of the refinement of grains. Now, in accordance with the Wettability and Interface-Bonding is concerned, the SEM images confirmed that the AZ91D matrix, Si_3_N_4_, and WGP particulates have exhibited a superior adhesion, bonding-strength, binding-affinity, and interfacial-attraction. Efficient wettability, and interfacial-bonding are parameters responsible for contributing to strengthen mechanical strength and load-bearing ability. Now, in context with the mechanical characteristics is concerned, the tensile-strength, hardness, and fatigue-strength of the developed composites were substantially strengthened. The incorporation of Si_3_N_4_ and WGP particulates, which possessed optimum suitable wettability, enhanced the work-hardening as well as mechanical characteristics. Now, in reference with the corrosion performance is concerned, the assessment for the resistance to corrosion revealed that the weight-loss of the composite was comparatively lesser than that of the AZ91D-matrix. The development of the barrier of oxide shield or protective-layer covering on the surface have contributed to its resistance to corrosion. Now, in context with the tribological behaviour is concerned, the resistance to wear for the composite was significantly enhanced, as evidenced by its reduced rate of wear as well as friction coefficients. Harder phases have been a contributing factor to the raised wear resistance. Finally, in accordance with the XRD analysis is concerned, the presence of harder refractory phases, including α-Mg, Al_12_Mg_17_, SiO_2_, Si_3_N_4_, MgO, and CaO, was observed through the XRD analysis. The aforementioned phases provided substantial contributions to the strengthening of mechanical as well as resistance to wear characteristics.

Hence, the enhanced characteristics reported in the composites based on AZ91D-matrix might be ascribed to the grain-fineness mechanism, which includes the development of harder-phases, efficient wettability, and a consistent homogenous-distribution. The combined synergistic effect of inclusion of WGP and Si_3_N_4_ particulates, when combined with controlled manufacturing parameters, led to notable enhancements in mechanical characteristics, resistance to wear, and resistance to corrosion. The research underlines the significance of mechanisms concerning grain-refinement in customising the characteristics of magnesium-based composites to suit a broader-spectrum array of applications.

### Number of grains with Grain fineness mechanism:

Upon microscopic examination, it became apparent that WGP and Si_3_N_4_ were distributed homogeneously across the AZ91D-matrix. It is imperative that these reinforcing-particulates has been evenly-dispersed in order to initiate the grain-refinement process.

The incorporation of 7.5% Si_3_N_4_, and 1.5% WGP particulates has significantly strengthened the material’s mechanical characteristics. The enhanced mechanical characteristics can be attributed to the refined structure of the grains.

The process of refinement of grains is accomplished through the modification of the α-Mg sizes of grain-particulates within the AZ91D-matrix. The aforementioned enhancement can be attained by means of the adequate or appropriate distribution of reinforcing-additives, efficient wettability, and the development of harder-phases throughout the fabrication of the composite^42^.

The incorporation of WGP as well as Si_3_N_4_ reinforcing-particulates culminated to a rise in tensile strength, demonstrating a positive favorable correlation among the concentration of reinforcing as well as mechanical characteristics. Nevertheless, as the wt% of reinforcing-additives raised, thereby, the ductility has been reduced, underlining the significance of refinement of grains when assessing the composite’s performance.

Furthermore, the Grain-count (n) per square inch at 500× magnification for AZ91D + 1.5%WGP + 7.5%Si_3_N_4_ composites was determined using Eq. (1).

1 $$n={2}^{(G-1)}$$

where n = the number of grains, G = grain size number ASTM.

2 $$G = [ - 6.644\log_{10} \left( {l_{\alpha } } \right)] - 3.28$$

where $$l_{\alpha } = \frac{{V_{v\alpha } \left( {L_{T} } \right)}}{{N_{\alpha } }}$$.

Here V_vα_ = fraction of α phase (volume), L_T_ = length/magnification (test line), N_α_ = grains intercepted (number),

Now,

$$\begin{aligned} & l_{\alpha } = \frac{{0.85 \times \left( {485/500} \right)}}{94}, \\ & l_{\alpha } = 0.00{87} \\ \end{aligned}$$

Now again G from Eq. (2),

$$\begin{aligned} & G = \left[ { - 6.644\log_{10} \left( {0.0087} \right)} \right] - 3.28 \\ & {\text{G}} = {1}0.{4}0 \\ \end{aligned}$$

Now from Eq. (1)

$$\begin{aligned} & n = 2^{{\left( {10.40 - 1} \right)}} \\ & n = 2^{{\left( {9.40} \right)}} \\ & n = 675.58 \\ \end{aligned}$$

The examination of a material’s microstructure at a 500× magnification has uncovered a significant revelation, shedding light on the intricacies of a composite material comprising AZ91D-alloy, 1.5%WGP, and 7.5%Si_3_N_4_. This detailed analysis has unveiled a precise and noteworthy observations, the material exhibits a remarkable density with 675.58 grains per square inch. The microstructural examination serves as a crucial tool in understanding the composition and properties of materials at a microscopic level. In the case for the AZ91D-alloy composites with WGP and Si_3_N_4_ particulates, the higher magnification has allowed for the identification and quantification of individual grains within the material. The specific grain count of 675.58 per square inch not only provides a quantitative measure but also hints at the material’s overall structural integrity and potential performance.

### Mechanical characteristics of fabricated composites

**Figure 18:**
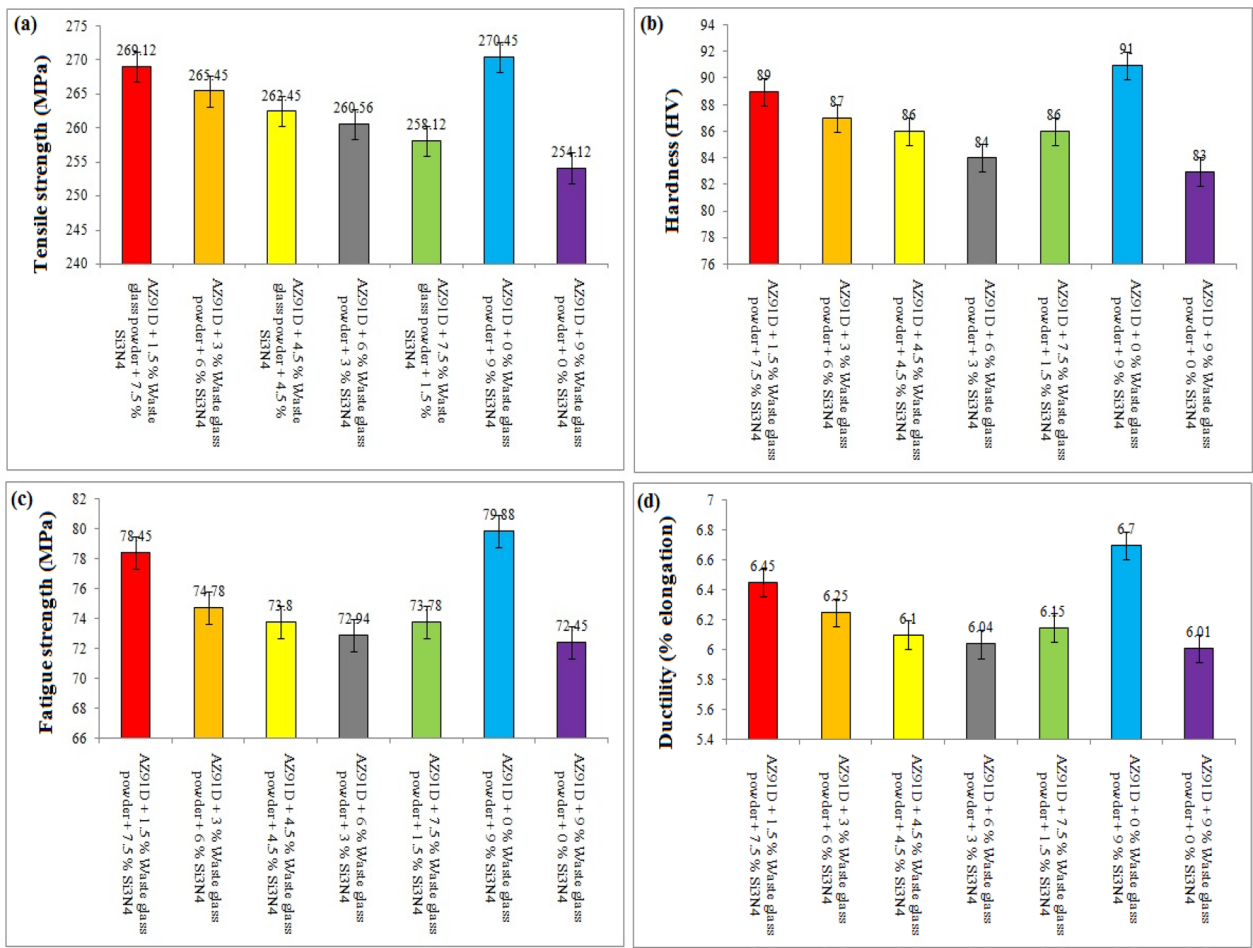
Analysis of (**a**) “Tensile Strength”, (**b**) “Hardness”, (**c**) “Fatigue strength”, (**d**) “Ductility” of the developed composites with distinct formulations.

Tensile strength was reported to be 269.12 MPa in the current experiment for the A1 formulated composite sample (Fig. 18a). Results, however, have also demonstrated that the tensile strength for the A6 formulated composite sample has considerably increased with the inclusion of 9-percent of Si3N4. The tensile strength has additionally reduced when WGP wt% in “AZ91D/7.5wt%Si_3_N_4_ composites” has increased. During tensile testing, the reinforcements with the formulation of developed sample A1, i.e., “(1.5%WGP + 7.5% Si_3_N_4_)” has significantly increase the work-hardening of the composite material. In the AZ91D matrix, this rise in work-hardening is also considerable at “1.5%WGP + 7.5% Si_3_N_4_”. After the incorporation of “reinforcing-constituent particulates” with “1.5%WGP + 7.5% Si_3_N_4_” to the AZ91D-matrix, the composite’s tensile strength escalated by 12.13 percent. In addition, the inclusion of “7.5% Si_3_N_4_” as “reinforcing-constituent particulates” to the AZ91D-matrix has raised the composite’s tensile strength by 12.68 percent. Figure 18b has summarized the computed hardness values for the various compositions for A1 formulated composite sample. With the escalation in “WGP” up to 1.5% weight percent in “AZ91D/7.5% wt. Si_3_N_4_ composites”, the hardness of magnesium-alloy has raised. The hardness for the A6 formulated composite sample, however, has exhibited a remarkable outcome, according to the analysis. Figure 18c has depicted the composite material made of magnesium’s fatigue strength. The weight-fractions of “reinforcing-constituent particulates” with “1.5%WGP + 7.5% Si_3_N_4_” have a notable impact on fatigue strength. Results revealed that the “1.5%WGP + 7.5% Si_3_N_4_” in the “AZ91D-matrix” has enhanced the fatigue strength by roughly 57.84%. The findings reported that by increasing the weight percentage of “WGP”, and “Si_3_N_4_”, the ductility of AZ91D was reduced as unveiled from the Fig. 18d. When compared to AZ91D-matrix, the composite material has unveiled lesser plastic deformation as its ductility declines. The ductility of “AZ91D + 1.5%WGP + 7.5% Si_3_N_4_ composites” is merely 7.85% lesser than “AZ91D-matrix material”. Understandably, the weight percentage of reinforcement, developed porosity, reinforcement features, grain refinement, etc., greatly improved the mechanical properties of composites made of magnesium. Strength, however, primarily depends on grain fineness^42,43^. The microstructure of the composite is thoroughly homogenized and enhanced through building the composite employing the vacuum pump, as seen in the SEM image (Fig. 14a–c). According to the Halle-Petch connection, the composite’s yield strength increased as grain size decreased. Second, during tensile deformation, the matrix material is transported together with the reinforcement particles that have the required wettability, improving the mechanical characteristics of the composite. Thirdly, another significant factor that affects the ductility and strength of the Mg-based composites is texture^43^. During the development of a Mg-based composite using waste glass and Si_3_N_4_, the basal texture with Schmidt factor evolved^12^.

#### Tensile-fractography analysis of developed composites

**Figure 19:**
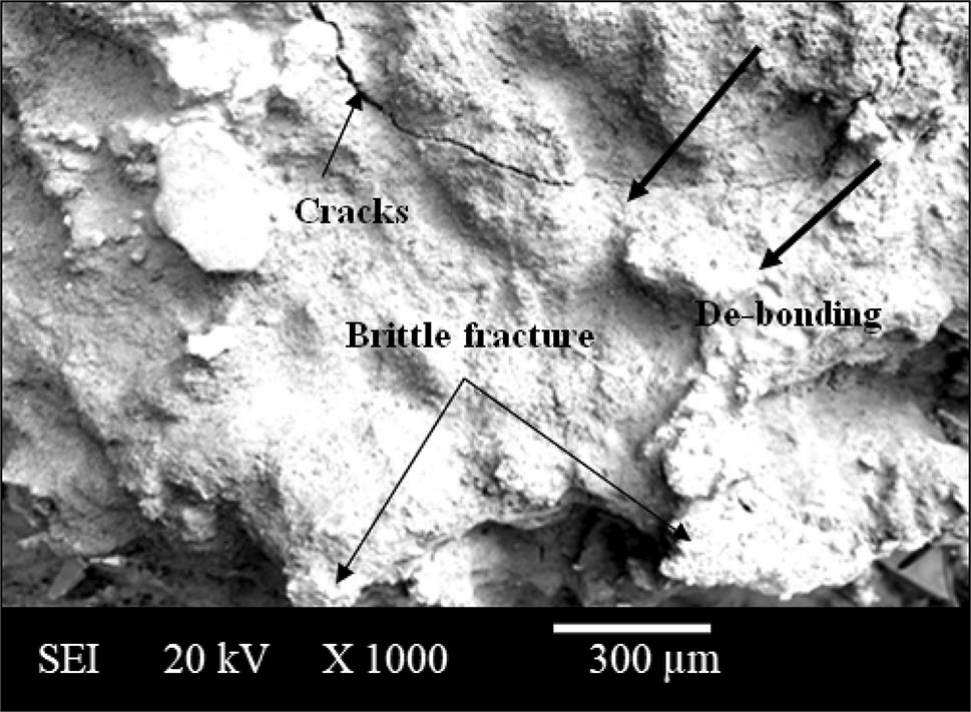
SEM image of Tensile-fractured surface of AZ91D + 1.5%WGP + 7.5% Si_3_N_4_ representative composite sample.

In accordance with the tensile-fractography examination for the developed composites has demonstrated the brittle fracture failure, including apparent cracks and debonding phenomena, as depicted from the Fig. 19. These findings have offered the significant insights into the failure mechanisms for the fabricated AZ91D composites. As the brittle fractures can be distinguished by the absence of substantial plastic-deformation prior to failure^40–43^. In AZ91D Mg-alloy, the presence of numerous parameters, including reduced ductility and raised stress-concentration, may eventually lead to the occurrence of brittle fractures characteristic in the developed AZ91D + 1.5%WGP + 7.5%Si_3_N_4_ composites. In addition, the AZ91D-alloy are highly vulnerable to cleavage-fracturing as a consequence of their hexagonally close-packed (HCP) structure of the crystals. When under tension, the planes of cleavage may propagate across the crystal lattice structure which produces fractures that are brittle on the surface of fabricated AZ91D + 1.5%WGP + 7.5%Si_3_N_4_ composites. The combined effect of stress-concentrations as well as lower-ductility may culminate to the propagation of planes of cleavage throughout the crystal lattice structure.

Moreover, the cracks are continuous linear fractures that occur when imperfections or regions with significant stress-concentrations propagate throughout a material. These stress-concentrations have served as a crucial role during the initiation as well as propagation of fracturing-cracks. Cracks can start in locations where stress is concentrated, such as impurities, inclusions, or empty spaces within the AZ91D + 1.5%WGP + 7.5%Si_3_N_4_ composites. During a tensile test, the application of raising stress has caused cracks to propagate throughout the material, eventually resulting in fracture. The application of tensile-force exacerbates the propagation of these cracks throughout the material.

Additionally, within the AZ91D + 1.5%WGP + 7.5%Si_3_N_4_ composites, debonding may arise as a consequence of insufficient bonding or adhesion among the matrix (AZ91D) and the reinforcing-phases (WGP, and Si_3_N_4_). Debonding of materials might occur caused by parameters involving inadequate wetting or the existence of oxides near surfaces. This has a potential to reduce the material’s strength, structural integrity and furthermore have an implication on the mechanical characteristics.

Although, the tensile-fractography microstructural examination might potentially reveal the existence of grain boundaries, which function as spots for the initiation of cracks or nucleation. The arrangement, dispersion, structural-layout, and positioning of grains might impact the fracture characteristics of the material. The particulate-agglomeration or clustering has affected the crack trajectory paths. Furthermore, the incorporation of reinforcing-particulates, i.e., WGP, and Si_3_N_4_, could potentially affect the path of cracks, causing crack-deflection or splitting or branched out, which in turn affects the performance or complete fracturing behavioral patterns during tensile-loading. Porosity, if exists, can serve as spots for stress-concentration as well as ultimately resulting in the initiation, emergence or formation of cracks. Hence, the examination of the tensile-fractured surface of the manufactured composites (AZ91D + WGP + Si_3_N_4_) unveils indications of brittle fractures, noticeable observable cracks, and debonding.

Moreover, the composite comprised of 9% Si_3_N_4_ as well as 0% WGP revealed favourable promising findings pertaining to its mechanical, corrosion, and tribological characteristics. The AZ91D Mg-based composites exhibited enhancements in microstructure, durability, resilience, toughness, mechanical-strength, resistance to abrasion, and protection against corrosion as an outcome of the Si_3_N_4_ and WGP reinforcing-particulates blend combination. Prospective applications in industries where such superior characteristics are desired have been proposed by the findings.

### Corrosion behavior of fabricated composites

**Figure 20:**
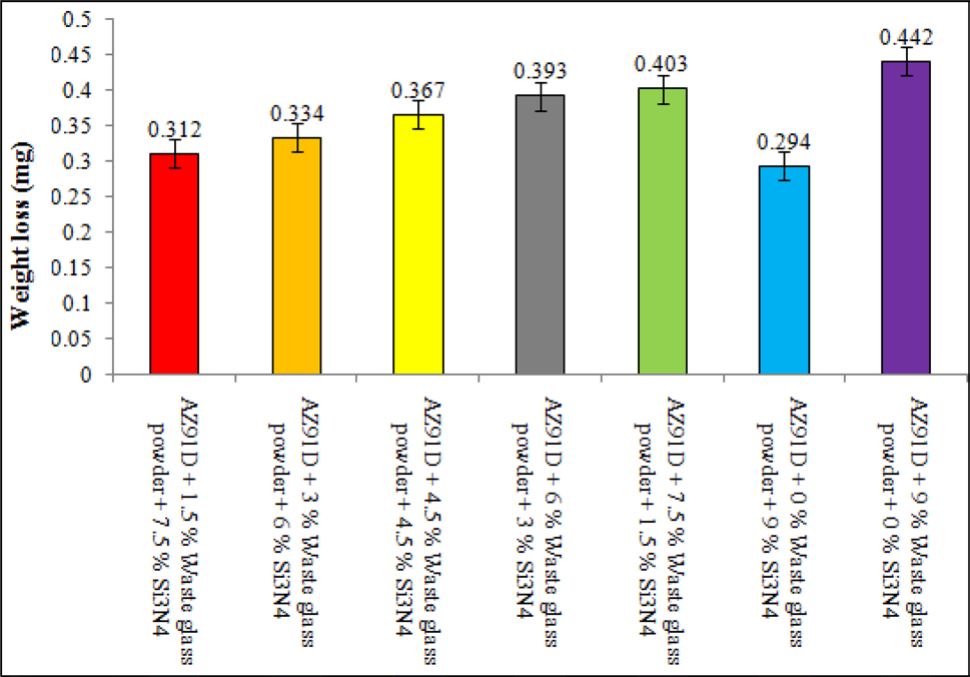
Corrosion weight loss of the developed composites with distinct formulations.

In 3.5 weight percent of NaCl corrosive-media for 120 h, the corrosion behaviour of the WGP, and Si_3_N_4_-reinforced AZ91D-based composites has been identified. The weight loss of the sample composition for A1 formulated composite was discovered to be 0.312 mg. Nevertheless, the weight loss for A6 formulated composite sample was found to be 0.294 mg as reported from Fig. 20. Compared to Si_3_N_4_, the presence of silica in waste glass is far more reactive in an acidic environment. However, the development of an oxide surface raises the probability that the surface has reacted with the environment, which has raised the possibility that corrosion has developed^40–43^. The influence of two key parameters is responsible for the corrosion µresistance and self-effacing resistance of magnesium and magnesium alloys. The high electronegativity of magnesium prevents corrosion when there is an oxygen shortage because cathodic water declines at a negative potential as a counteraction^40–43^. Due to the hydroxide layers’ solubility, the self-effacing shielding characteristic or uniqueness of the films under wet conditions evolved on the surface^13^. MgO is enhanced by the instantaneous interactions of magnesium with oxygen at room temperature. In dry settings, this reaction was seen to occur without any water. The weight-loss for the composite comprised of 0% WGP and 9% Si_3_N_4_ within AZ91D-matrix has been reported, and the corrosion-related behaviour of the composite has been assessed under a 3.5% NaCl solution. As it has been noticed that the reactivity of SiO_2_ in WGP particulate in other formulations has been heightened or strengthened when subjected to acidic surroundings. Furthermore, the corrosion characteristics exhibited by the WGP particulate have been impacted by the breakdown of the oxide barrier, protective oxide covering layer, or protective oxide shield when chloride ions have been present. Incorporating both the WGP as well as Si_3_N_4_ particulates in combination facilitates the development of a protective shielding oxide-layer; however, the inclusion of chloride ions into the mix accelerates the corrosion process.

**Figure 21:**
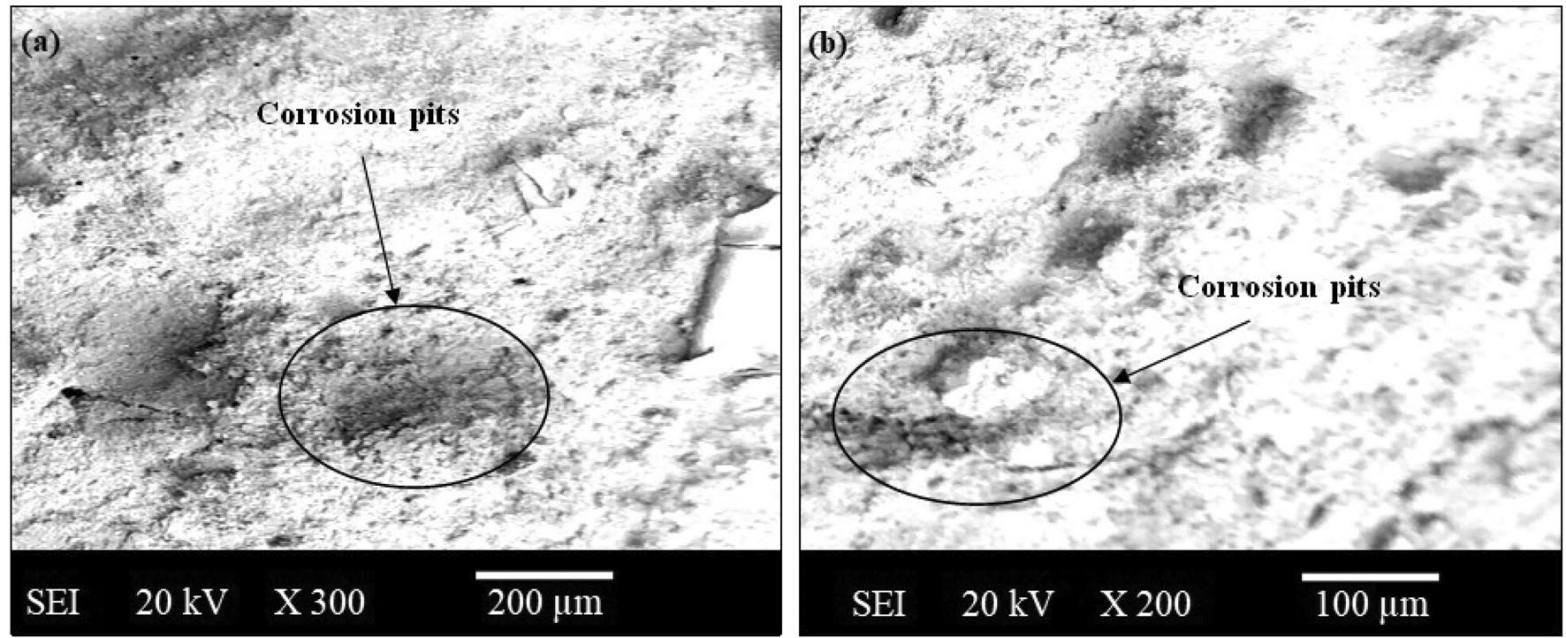
SEM micro-images of corroded-surface for “AZ91D + 1.5% WGP + 7.5% Si_3_N_4_ composites”.

The existence of NaCl solution has accelerated the corrosion. Chloride ions have the potential to cause the protective oxide-layer that usually forms on AZ91D-alloy to break down in an aqueous environment. As a result of this breakdown, pits and corrosion products commence to form on the surface and the underlying metal is exposed to the corrosive environment^43^. SEM imaging have illustrated the depressions or craters as the corrosion pits on the sample’s surface. SEM images has been employed to analyze and quantify the topography of the pits as well as their size, depth, and dispersion^42,43^. Due to variations in “surface morphology”, and “composition”, distinct “electron emission” outcomes in the contrast among the pit bottoms and the surrounding material^14,15^. As a consequence of “localized surface attack” brought on by “chemical interactions” with the “corrosive environment”, the “corrosion pits” have been developed as exhibited in the Fig. 21a,b.

### Tribological behaviour of fabricated composites

**Figure 22:**
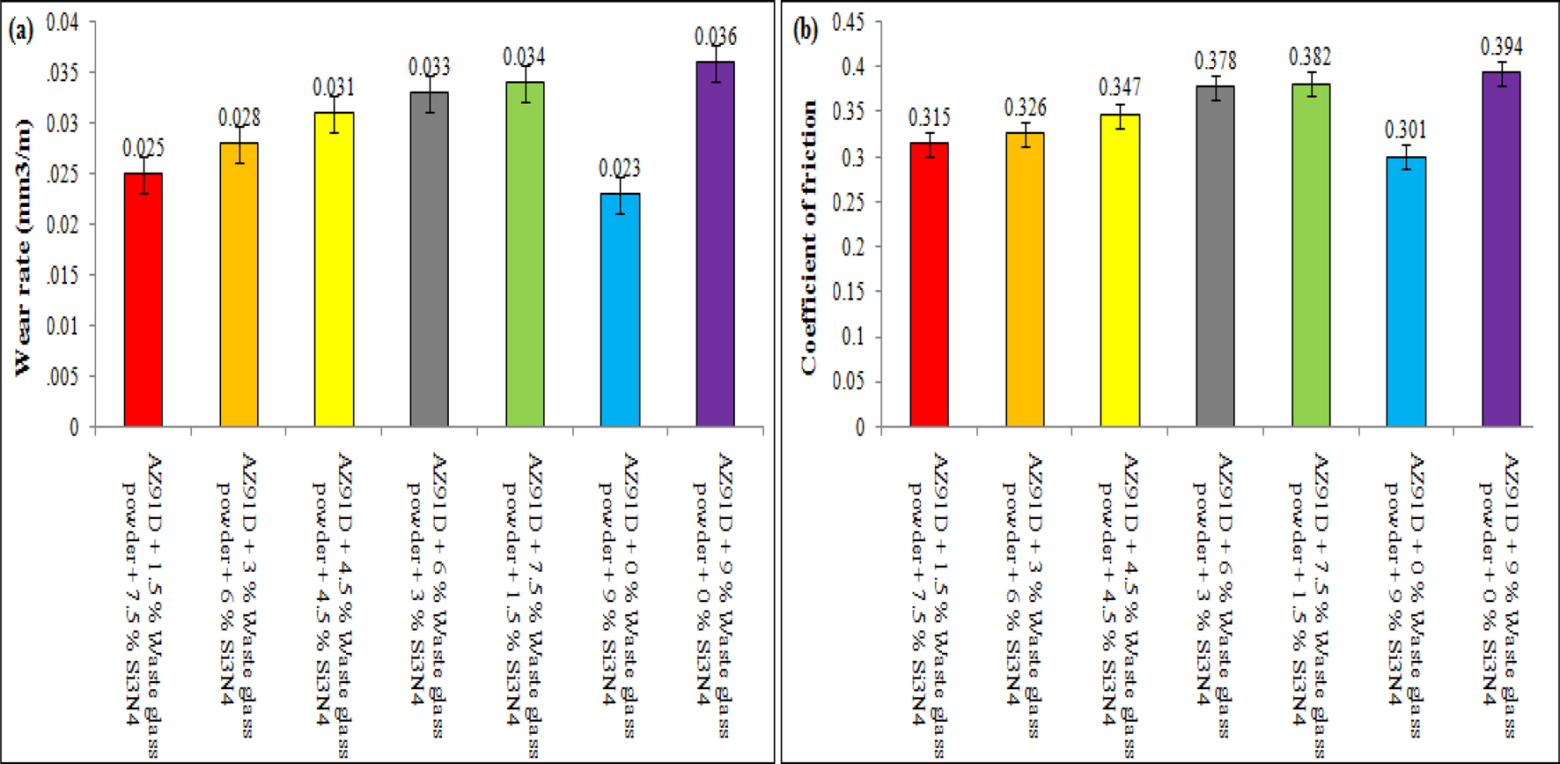
(**a**) Wear behavior, (**b**) Friction behavior of the developed composites with distinct formulations.

The present investigation has examined the wear and friction behaviour of a magnesium-based composite with distinct combinations of “WGP”, and “Si_3_N_4_”. Cylindrical pins (Mg-based composite with various mixes of WGP and Si_3_N_4_) with a height of 10 mm and a diameter of 6 mm were employed for the Pin on Disc testing. Each pin’s flat end made touch with the turning disc. The discs were 63 mm in diameter and 6 mm thick, and they were made of cast iron with an average surface roughness of 2 µm and a hardness of 235 HV10. On the basis of the investigation from the pilot-run, the wear-test factors have been selected. The wear and friction behaviour of a “magnesium-based composites” with distinct combinations of “WGP”, and “Si_3_N_4_” is shown in Fig. 22a,b. At 5 N loads, 2 m/s sliding speed, and 1000 m of sliding distance, the “wear-rate”, and “coefficient of friction” for the developed A1 formulated composite sample were discovered to be 0.0025 mm^3^/m and 0.315, respectively. Findings have indicated that the inclusion of “reinforcing-constituent particulates” with “1.5%WGP + 7.5% Si_3_N_4_” combination has considerably increased the developed composite’s resistance to “wear-rate”^16,17^. The enhancement in the “wear-resistance” of the “base-matrix material (AZ91D)” was brought about by the development of hard phases such as “α-Mg”, “Al_12_Mg_17_”_,_ “SiO_2_”, “Si_3_N_4_”, “MgO”, and “CaO” phases (Fig. 24).

A composite with the formulated-composition of 0% WGP + 9% Si_3_N_4_ within AZ91D-matrix composites has been noted for its slight contributions to the enhancement of mechanical characteristics, structural-integrity, and the enhancement of resistance to wear. XRD analysis has revealed an additionally evidence that the inclusion of Si_3_N_4_ has contributed to the development of harder-phases^40–42^.

All in all, with reference to the formulated composite compositions, a substantial enhancement has been unveiled in the tribomechanical, corrosion and other characteristics upon the incorporation of Si_3_N_4_ at an optimal wt% of 7.5% and WGP particulates at 1.5%.

**Figure 23:**
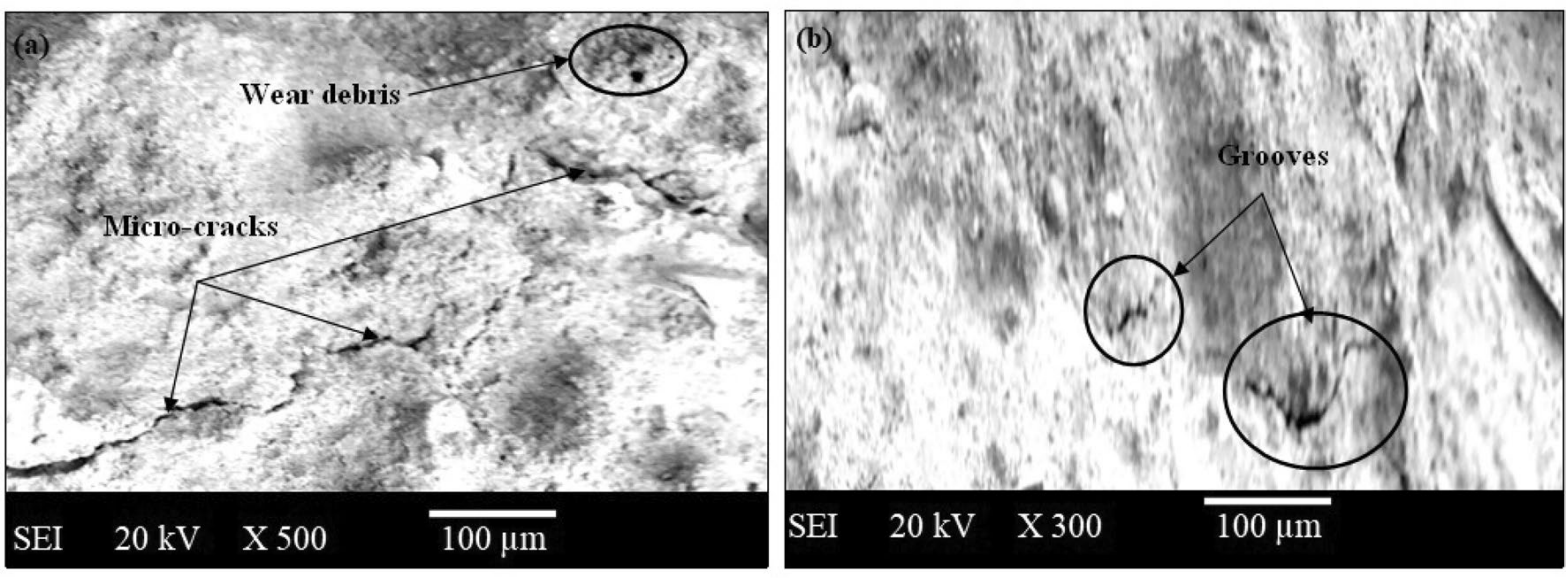
SEM micro-images of wear-surface for “AZ91D + 1.5% WGP + 7.5% Si_3_N_4_ composites”.

Small particles or debris can be produced during wear processes when the material is subjected to friction and abrasion as evident from the Fig. 23a,b. These granules may break free from the surface and accumulate nearby. These debris fragments has appeared as surface features with irregular forms in SEM images (Fig. 23a,b).

An indication of abrasive-wear may be the existence of profound grooves and debris. As a result of the countersurface’s asperities or hard particles cutting into the softer material during abrasive wear, material is removed and grooves are formed^40–43^. The abrasive wear observed in this instance may be caused by Si_3_N_4_ particles or other hard components in the composite^18,19^.

The microcracks observed in the Fig. 23a,b point to the occurrence of fatigue or repetitious loading-conditions. Microcracks can commence and spread as a result of “stress concentrations” as the material is subjected to “cyclic-stresses”. The WGP particulates may present heterogeneous-regions into the AZ91D-matrix, producing “stress-concentration spots”, and promoting the formation of microcracks.

In addition, the “delamination” may take place if the composite has “porous interfaces” between the various phases (“AZ91D-alloy”, “WGP”, “Si_3_N_4_”). Layers can separate along “porous interfaces” when “mechanical stresses” are present, a process known as “delamination”. This may result in the “accumulation of debris”, and “accelerate wear”^14,15^.

In response to “applied loads”, the AZ91D-alloy portion of the composite can deform plastically. The “surface topography” may change and grooves may form as a result of this deformation.

The “Si_3_N_4_”, and “WGP” can be added to the AZ91D-matrix to change its “mechanical characteristics”, and “wear pattern”^20,21^. The hard ceramic-particulates (Si_3_N_4_) may raise the composite’s “hardness”, and “resistance to wear”, while the “WGP” may affect “fracture toughness”, and possibly aid in “crack initiation”, and “propagation”^40–43^.

### XRD analysis of representative composites

**Figure 24:**
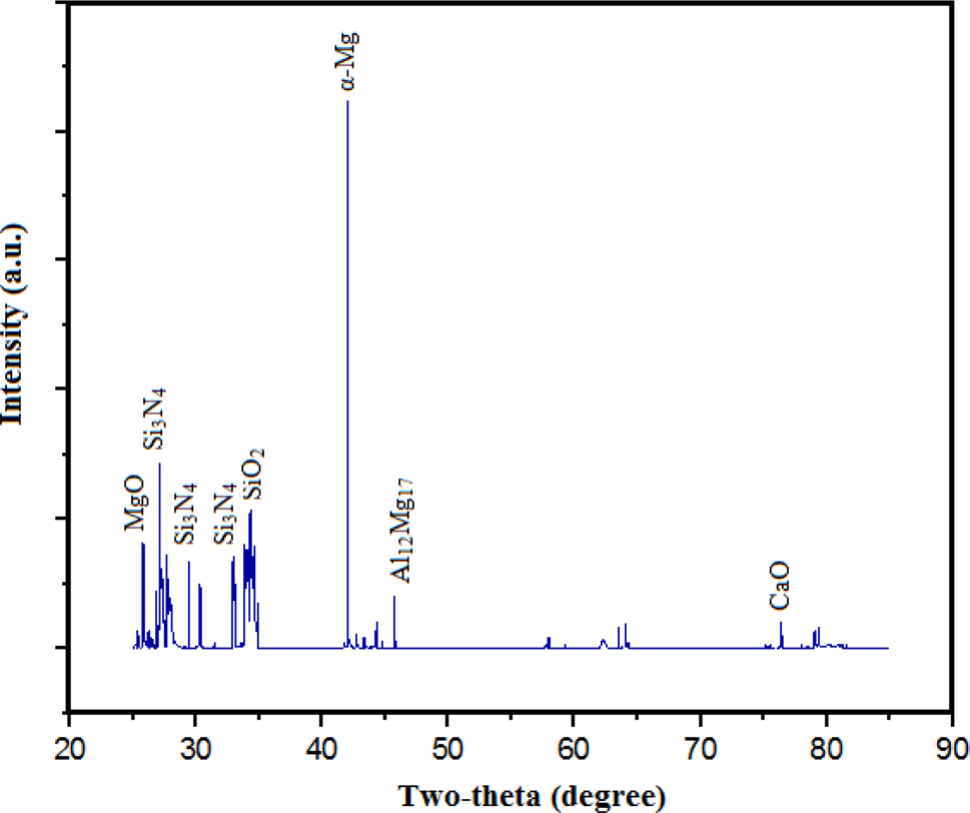
XRD analysis of “AZ91D + 1.5% WGP + 7.5% Si_3_N_4_ composites”.

An in-depth understanding of a material’s “physical characteristics”, “chemical composition”, and “crystallographic structure” can be obtained through “X-ray diffraction (XRD) analysis”. It is based on a “productive-interference” of a “crystalline specimen”, and “monochromatic X-rays”. The “XRD behaviour” of produced composite has apparently been observed. The “surface behaviour”, “wear”, and “mechanical behaviour” of the developed composite are represented by the evolved phases inside the composite^14,15^. Figure 24 has demonstrated that the composite material has the phases “α-Mg”, “Al_12_Mg_17_”_,_ “SiO_2_”, “Si_3_N_4_”, “MgO”, and “CaO” phases. Peaks that are clearly defined and intense point to a higher degree of crystallinity and to well-ordered atomic arrangements within the phases. Broader and lesser intense peaks might be a sign of amorphous phases or a decrease in crystallinity^20^. Understanding their atomic arrangements within the composite material is also aided by the evaluation of the crystal-structures of “α-Mg”, “Al_12_Mg_17_”, “SiO_2_”, “Si_3_N_4_”, “MgO”, and “CaO” phases^43,44^. The role of identified phases through XRD Analysis has been elucidated as follows. Firstly, the crucial primary phase of the AZ91D magnesium alloy is α-Mg. The identification regarding the composite’s comprehensive microstructure as well as its mechanical characteristics has been substantially dependent on its presence. Alpha-magnesium grains enhance the strength, durability, resilience, and excellent castability of the composite. When examined through optical microscopy, the particulate size promises significant insight pertaining the circumstances of solidification. The presence of clearly defined distinct peaks associated with the α-Mg phase has been confirmed through XRD analysis, implying a significant degree of crystallinity with properly organized, and structured pattern of atomic-arrangements. An optical microscope illustrates that the existence of larger α-Mg grains has been correlated with a rise in mechanical strength. The material’s mechanical characteristics are significantly impacted by the dimensions, arrangement, and dispersion structure layout of α-Mg grains. Variations in methods for post-processing as well as solidifying circumstances may be suggestive of larger grain particulates. Now, the second phase, Aluminium magnesium intermetallic-phase (Al_12_Mg_17_) is concerned, this intermetallic-phase Al_12_Mg_17_ has a capability to impact corrosion’s behaviour as well as mechanical characteristics of the composite. The appearance of Al_12_Mg_17_ might potentially strengthen the resistance to wear. In order to optimally maximise the positive implications, its dispersion as well as interaction with other phases are, nevertheless, crucial. Now, the third phase Silicon Dioxide (SiO_2_) is concerned, as SiO_2_ is a phase that has been apparently generated from the waste glass particles. It has served as a pivotal contribution in effecting the wear as well as mechanical characteristics of the composite’s material. The inclusion of SiO_2_ phase within the composite material might strengthen its resistance to abrasive-wear. SiO_2_ has the potential to enhance the material’s hardness and resistance to wear. In combination with various other phases, its continued existence has influenced the composite’s overall resistance to wear, deformation or damage or degradation, deterioration. Now, the fourth phase Silicon Nitride (Si_3_N_4_) is concerned, as Si_3_N_4_ has been incorporated to the composite as a secondary reinforcing material, thereby enhancing its resistance to wear as well as wettability, structural integrity, strengthens the adhesion bonding to the AZ91D-matrix, functionality, resilience, durability, and performance in mechanical terms. Si_3_N_4_ has possessed the exceptional mechanical characteristics and is stable under thermodynamic conditions. Its crystalline phases, including beta-Si3N4 and alpha-Si3N4, are contributing to the hardness as well as abrasion resistance of the composite. Numerous crystal structure forms or crystallographic forms, and phases of crystalline structure of Si_3_N_4_ (alpha, beta, hexagonal) are contributing towards the composite’s general characteristics. It significantly influences the enhancement of mechanical characteristics as well as resistance to wear. Now, the fifth phase Magnesium oxide (MgO) is concerned, as the formation of MgO is generally attributed to the chemical reaction that occurs among magnesium and oxygen during the process of manufacturing or when subjected to the environmental surroundings. MgO phase has potentially strengthened the composite’s resistance to corrosion. MgO phase is well-known for its chemically stable characteristics, as well as its elevated melting point. Thermal as well as chemical resistant characteristics for the composite may be impacted by incorporating it in the material. The characteristics of MgO consist of the potential prospective to enhance resistance to corrosion as well as determining the implications of composite’s stability in general throughout different environmental circumstances. Now the next phase Calcium oxide (CaO) is concerned, as CaO, which is comparable to MgO, can be generated as a consequence of chemical reactions that occur during the processing stage. It might impact the characteristics of the composite. The resistance to corrosion as well as chemically stable properties for the composite might be influenced by the incorporation of CaO.

Thus, the XRD analysis has confirmed that the synergistic combined blend effect of these phases (α-Mg, Al_12_Mg_17_, SiO_2_, Si_3_N_4_, MgO, and CaO phases) have substantially affected the comprehensive performance and effectiveness of the AZ91D magnesium-based composites. The phases that have been identified correspond with the enhancements in mechanical characteristics, resistance to wear, and corrosion behaviour that were reported in the study’s findings.

### Desired applications of vacuum stir casted AZ91D + 1.5%WGP + 7.5%Si_3_N_4_ composites:

Broader spectrum and potentially influential across numerous industries are engineering applications of AZ91D Mg-MMC’s reinforced with Si_3_N_4_ and WGP particulates^42–44^. Based on the information offered and the findings and characteristics it reveals, the following are several significant applications:

#### Automotive sector

1. *Weight Reduction* Mg-based composites are well-known for their capacity to reduce weight. The mechanical characteristics (raised tensile-strength and resistance to wear) of developed composites have been enhanced through the incorporation of Si_3_N_4_ as well as WGP, rendering them well-suited for light-weight automotive parts. This, in turn, leads to enhanced engine performance, energy efficiency, energy savings, vehicle performance, environmental sustainability, fuel efficiency and reduced emissions.
2. *Wear Resistance* The utilisation of composite materials consisting of 1.5% WGP and 7.5% Si_3_N_4_ effectively improves wear resistance. As a result, these materials are well-suited for applications involving components that are susceptible to friction and wear, including engine parts, brake components, and gearbox systems.

#### Aerospace applications

1. *Weight savings* In the aviation sector, magnesium composites are significant paramount for minimising the total weight of aircraft as a result of their light weight. Steel brackets, housings, and structural components, underlying support structures or foundation part elements are instances of components whose mechanical characteristics have the potential to be enhanced.
2. *Resistance to Corrosion* Material resistance to corrosion is a crucial requirement or vital requirement in aviation applications as a result of numerous environmental conditions which materials have been subjected towards

#### Industrial equipment machineries

1. *Components Resistant to Wear* The wear resistance exhibited by the composites, especially when 1.5 percent WGP and 7.5 percent Si_3_N_4_ are incorporated, rendering them viable materials for abrasive wear-resistant machinery parts, thus prolonging the functional operational service life of such parts.

#### Electronics and electrical components

1. *Mechanical Strength* The strengthened mechanical characteristics of the composites, like, raised tensile strength, modulus, and hardness, render them advantageous in the manufacture of durable electronic and electrical components that require exceptional physicomechanical strength.

#### Building and construction sector

1. *Structural Components* The structural parts are appropriate to such composites as a result of their durability, resilient to mechanical forces, strength, mechanically robust yet light-weight characteristics, providing them appropriate for usage in construction. Application possibilities have comprised of connectors, light-weight panels or boards or sheets, or support structures.

#### Manufacturing and tooling

1. *Wear-Resistant Equipment* The enhanced resistance to wear caused by abrasive agents exhibited by the composite material leads to this well-suited appropriate to be employed in the manufacturing of tools as well as machinery components. This could culminate to raised tool longevity, durability, lifespan, reliability, long-term viability, lifespan sustainability, and reduced maintenance expenses.

#### Structural components

1. Superior mechanical characteristics, like toughness, durability, resilience, fracture resistance, tensile strength and hardness, rendering the composites appropriate for usage in structure building frameworks.
2. Corrosion resistance and resistance to wear are desirable characteristics that can be employed in marine settings for shielding or protecting the structural components against environments that are corrosion-prone.

#### Military and defence applications

1. When reduction in weight tends to be of the nature, magnesium composites possess the potential to be valuable in applications concerning defence and military equipment owing to their light weight as well as their superior durability.

Furthermore, the incorporation of WGP, as well as Si_3_N_4_ as reinforcing agents within AZ91D Mg-MMCs offers a spectrum of realistic engineering perspectives characterised by enhanced mechanical, tribological, and corrosion characteristics. The following are several pivotal consideration aspects concerning practical applications as well as significance of the findings:

#### Enhanced mechanical characteristics

The incorporation of Si_3_N_4_ at a weight percentage of 7.5% as well as WGP at a weight percentage of 1.5% has substantially raised the tensile strength of AZ91D magnesium alloy. A rise of 12.13 percent in tensile strength indicates enhanced mechanical performance, resistance to deformation or wear, strength, durability, stability, structural-strength, and functionality.

The developed composites have revealed enhanced characteristics in terms of hardness, toughness, durability, strength, fatigue strength, and corrosion resistance. The efficient mechanical characteristics were enhanced through the emergence of hard phases, namely α-Mg, Al_12_Mg_17_, SiO_2_, Si_3_N_4_, MgO, and CaO, which were aided by the blend combination of WGP and Si3N4.

#### The resistance to wear

A substantial reduction in both the wear rate and coefficient of friction was observed in AZ91D composites comprising 1.5% WGP as well as 7.5% Si_3_N_4_, signifying an enhancement in resistance to wear. The existence of wear caused by abrasive debris as well as averting or preventing the occurrence of wear have been both consequences for the existence of harder phases.

The enhanced resistance to wear can be attributable to the development of harder ceramic or refractory phases, which include α-Mg, Al_12_Mg_17_, SiO_2_, Si_3_N_4_, MgO, and CaO. Depending on the material’s resistance to microcrack or tiny-fractures formation along with abrasive wear, the composite appeared to be well-suited or ideally appropriated/suitable for applications requiring sliding as well as abrasive contact.

The appearance of micro-cracks or tiny fractures on the worn-surfaces could potentially signify that the composite was subjected to cyclic loading conditions. The long-term performance, reliability, stability, viability, durability, and long-lasting resilience of the reinforcing in the composite can be ascribed to its capacity for withstanding mechanical stresses.

#### Anti-corrosion characteristics

The corrosion assessment was performed for the composites within a corrosive environment comprising of a 3.5% NaCl solution. In comparison with additional formulation compositions, the weight loss of the composite comprising of 1.5 percent WGP as well as 7.5 percent Si_3_N_4_ was lower, exhibiting enhanced resistance to corrosion.

The corrosion of the AZ91D matrix has been mitigated by the development of a protective oxide barrier or shielding layer coating on the surface, which was made more effective or facilitated through the inclusion of Si_3_N_4_ and WGP.

#### Microstructural assessment

The homogeneous evenly dispersal for WGP and Si_3_N_4_ in the AZ91D matrix has been demonstrated by microscopic examination, revealing that processing, blending, and dispersion have been carried out efficiently. It is crucial for attaining consistent mechanical characteristics throughout the composite by this uniformly even distribution.

The relatively continuous separation of WGP and Si_3_N_4_ particulates reported by the SEM images has indicated that the manufacturing technique was effective in attaining a uniform or evenly dispersion.

#### Wettability and the adhesion-bonding strength at the interfaces

The findings of the wettability study revealed that the AZ91D matrix as well as the reinforcing particles (WGP and Si_3_N_4_) developed durable/resilient interfacial bond adhesion strength. The durable interface in concern strengthens both the capacity of bearing loads as well as the overall performance functionality, resilient, efficiency, durability, strength, structural integrity, and mechanical performance.

The formation of chemical bonds or existence of chemicals-interaction has been demonstrated by the emergence of a reactive Si–Al–O–N layered at the interface that exists between Si_3_N_4_ as well as AZ91D; this enhanced the adherence bond-strength among the two materials.

#### Structural analysis

The X-ray diffraction analysis has affirmed the presence of several phases along with provided insightful perceptive comprehension regarding the crystallographic structure, crystallographic arrangement, or the structural of a crystal for the developed composites. A higher degree of crystallinity was apparent by apparent well defined distinct peaks, which contributed to the enhanced mechanical and physical characteristics.

The existence of clearly prominent discernible well defined peaks suggests that the material possesses a substantial level or higher degree of crystallinity or crystalline-structure, along with ordered atomic-arrangements, both of which strengthen the composite material’s overall performance.

As an outcome of incorporating Si_3_N_4_ and WGP as reinforcement particulates to AZ91D magnesium composites, superior mechanical, tribological, and corrosion characteristics have been accomplished. In automotive components, aerospace structures, and other high-performance engineering applications where raised strength, resistance to wear, and resistance to corrosion are crucial, the synergistic blend combination of these additives possesses real-world applications.

## Conclusions

In order to prevent the unfavourable effects of oxidation, this study has utilised recycled WGP, and Si_3_N_4_ as reinforcing-particulates within AZ91D Mg-based composites fabricated through vacuum stir casting. The AZ91D-matrix has comprised an evenly-distribution of WGP and Si_3_N_4_ reinforcing-additives, as evidenced by microscopic analysis. By contemplating the microstructural morphology, tribological, corrosion, and physicomechanical characteristics of the AZ91D-based composites, the subsequent conclusive findings have been inferred as follows:

1. The AZ91D Mg-matrix exhibited the uniformly evenly-distribution of WGP and Si_3_N_4_ reinforcing-particulates, as revealed by the microstructural analysis. Analysis of the reinforcements’ wettability and interface adhesion-strength with the AZ91D-matrix materials has assured efficient dispersion.
2. The tensile strength of composites AZ91D/WGP/Si_3_N_4_ was enhanced by incorporating 1.5% WGP into AZ91D/7.5%Si_3_N_4_. The combination of AZ91D/9%Si_3_N_4_ exhibited the highest tensile strength among the selected compositions, far exceeding than the other remaining combinations. In particular, the tensile strength for the composite AZ91D/1.5%WGP/7.5%Si_3_N_4_ was 12.13 percent superior than that of the AZ91D-matrix base-alloy.
3. By incorporating 1.5% WGP reinforcing-particulates into AZ91D/7.5%Si_3_N_4_, the Mg-alloy’s hardness has been strengthened. The composite samples formulated with A6 exhibited superior hardness findings. In addition, the fatigue strength for AZ91D/1.5%WGP/7.5%Si_3_N_4_ raised by around 57.84%. However, the ductility exhibited a reduction in wt% of WGP and Si_3_N_4_ reinforcing-particulates.
4. The corrosion performance for the formulated composites with A1 and A6 under 3.5 percent NaCl for a duration of 120 h has revealed a weight-loss of 0.312 mg and 0.294 mg, respectively. The resistance to corrosion for WGP particulates has been noticed to be influenced by the reactive nature of silicon dioxide in an acidic atmosphere.
5. The EDS spectra-analysis have affirmed an evident occurrence of the respective Mg-peak, Si-peak, Al-peak, Ca-peak, and O-peak curves for the 1.5%WGP/7.5%Si_3_N_4_/AZ91D composites.
6. The tensile-fractography examination for the developed 1.5%WGP/7.5%Si_3_N_4_/AZ91D composites has exhibited the brittle fracture failure, including cracks and debonding phenomena.
7. The findings for pin-on-disc testing revealed that AZ91D, reinforced with 1.5%WGP/7.5%Si_3_N_4_, evidenced enhanced wear-resistance (0.0025 mm^3^/m) and a frictional coefficient of 0.315 when subjected to 5N load, a sliding-speed of 2 m/s, and a distance of 1000 m. The rise in resistance to wear has been ascribed to the formation of harder-phases, like α-Mg, Al_12_Mg_17_, SiO_2_, Si_3_N_4_, MgO, and CaO.
8. The composite has been demonstrated to be composed of α-Mg, Al_12_Mg_17_, SiO_2_, Si_3_N_4_, MgO, and CaO phases, as identified by XRD analysis. The existence of clearly defined and focused peaks within the phases revealed a higher degree of crystallinity as well as orderly structured atomic arrangements.
9. These developed composites are significantly potential candidates for industries which require light-weight, resistant to corrosion, and long-lasting durable, resilient or stable materials, including automobile and aviation sectors, owing to their enhanced resistance to abrasion as well as higher tensile strength.

## Future outlook

The research conducted on Si_3_N_4_/WGP/AZ91D based Mg-based composites that has offered the prospective avenues and potential opportunities for additional investigation in the realm of advanced materials. In an effort to enhance an understanding as well as application of AZ91D composites, additional investigation could potentially be pointed towards the subsequently identified resulting areas. The forthcoming prospective potential dimensions or scope has comprised:

1. *Optimisation of Reinforcements Ratio Analysis* Determine the optimal blend combination of weight proportions of WGP and Si_3_N_4_ to strengthen mechanical and wear characteristics by through analysing the implications of varying these weight percentages. In addition, examine the implications of varying processing factors-such as stirring-speed, stirring-duration, as well as preheating-temperature-on the overall performance of the composite material throughout its entirety with the objective to determine what circumstances are the most favourable for accomplishing superior reinforcing distribution. Moreover, conduct an analysis of alternative fabrication techniques, including extrusion as well as powder metallurgy, in order to assess their comparative effectiveness within the manufacturing of high-performance magnesium composites.
2. *Incorporation of Additional Reinforcements* Examine the prospective potential benefits of incorporating alternative reinforcements, such as carbon-based materials (e.g., graphene and carbon-fibres), in order to discover synergistic improvements in wear as well as mechanical characteristics.
3. *Microstructural Analysis* Technological advanced imaging approaches, including transmission electron microscopy (TEM), and AFM analysis could potentially be employed in order to attain an in-depth comprehension for the interfacial bonding adhesion-strength that occurs among the AZ91D-matrix and WGP as well as Si3N4 reinforcements.
4. *Heat Treatment Investigations* Examine the implications of various heat treatment methods on the mechanical characteristics as well as resistance to corrosion for the composite material in order to tailored or customised it to suit for distinctive applications.
5. *Environmental Implications* Examine the performance functionality, efficacy, effectiveness, or efficiency, and long-lasting durability of the composites over a prolonged periods of time when subjected under various circumstances in the environment, such as variations in temperature as well as corrosive agent exposure.
6. *Analysis for Processing Methods* Evaluate the implications on the alternate advanced manufacturing processes beyond vacuum stir-casting method on the matrix dispersion of reinforcing-particulates and mitigate the defects or imperfections in the developed composites. Prospective developments in techniques for manufacturing that are being explored include powder-metallurgy as well as additive manufacturing.
7. *Multi-Functional Composites* Multifaceted composites have the potential to be manufactured by incorporating additional reinforcement materials or nanoparticles. These composites would exhibit strengthened thermal conductivity, electrical conductivity, or additional desirable characteristics.
8. *Nanostructured Additives* This investigation seeks at examining the potential possibilities or applications of nanostructured additives, specifically Si_3_N_4_ as well as WGP, to significantly enhance the distribution characteristics within the AZ91D-matrix along with subsequently strengthen its mechanical characteristics.
9. *Multi-Scale Modeling* Multi-scale modelling ought to be adopted in order to simulate the composite’s behaviour or performance activity across various length scales or dimensions. This will give researchers an additionally comprehensive understanding concerning the material’s thermal as well as mechanical characteristics.
10. *Statistical analysis and Computational Modelling* Develop computational mathematical models that simulate the composite’s performance under varying loading-conditions; this will aid in the optimisation as well as prediction of mechanical characteristics. In addition, the incorporation of statistical analysis or mathematical modelling to further validate the reliability for the findings along with derive additional robust outcomes concerning the scope of the reported enhancements.
11. *Life Cycle Assessment (LCA)* To aid in the development of sustainable materials, perform a LCA technique aimed at assessing the adverse environmental consequences for both the manufacturing and disposal of such composites.
12. *Advanced Characterization Techniques* By employing advanced methods for characterization, including in-situ measurements, it can be accomplished to record instantaneous real time variations within the material as it undergoes mechanical testing or is subjected to the surrounding environment.

By contemplating such aspects, the further research studies and subsequent investigations may expand upon the present investigation while offering a valuable contribution to the progressive development of magnesium-based composites throughout a broader spectrum array variety of applications.

## Limitations of the present study

Although substantial advanced findings and progressive developments have been accomplished in the present research, it is nevertheless essential to understand the following limitations:

1. *Analysis of a Single Composition* The investigation was confined to a particular set of compositions; subsequent studies ought to take into account a broader spectrum array of compositions in order to ascertain the material’s versatility or resilience.
2. *Environmental Implications* An exhaustive evaluation regarding the environmental consequences, involving the consumption of energy throughout the manufacturing process along with final or eventual end-of-life disposal considerations, has been absent from the study.
3. *Simplified Corrosion-environmental Limitation* The corrosion activity was examined within a specified environment comprising 3.5% NaCl. The response to additional corrosive circumstances necessitates additional research.
4. *Theoretical Modeling* The investigation has limitations in theoretical mathematical or computational model frameworks that make predictions regarding the behaviour that was observed. Additional investigations are appropriate to take into account conceptual frameworks or mathematical-models in order to shed light on testing outcomes.

## Data Availability

The datasets generated and/or analysed during the current study are available merely within the manuscript.

## Abbreviations

WGP: Waste glass powder
Si_3_N_4_: Silicon nitride
AZ91D: Magnesium-based alloy
SEM: Scanning electron microscopy
MMC’s: Metal-matrix composites
XRD: X-ray diffraction
α-Mg: Alpha-magnesium, primary phase in the AZ91D matrix
Al_12_Mg_17_: Aluminum-magnesium phase
SiO_2_: Silicon dioxide
MgO: Magnesium oxide
CaO: Calcium oxide
Si–Al–O–N phase: Silicon aluminum oxynitride phase
NaCl: Sodium chloride

## Symbols with units

°C: Degree celsius
MPa: Megapascal
rpm: Revolutions per minute
N: Newton
m/s: Meters per second
mg: Milligram
wt%: Weight percentage
µm: Micrometer
HV10: Vickers hardness (10 kg load)
µresistance: Micro-resistance
A1, A2, A3, A4, A5, A6, A7: Codes for different composite formulations
